# Efficacy and Safety of Umbilical Cord-Derived Mesenchymal Stromal Cell Therapy in Preclinical Models of Sepsis: A Systematic Review and Meta-analysis

**DOI:** 10.1093/stcltm/szae003

**Published:** 2024-02-21

**Authors:** Christine Hum, Usama Tahir, Shirley H J Mei, Josee Champagne, Dean A Fergusson, Manoj Lalu, Duncan J Stewart, Keith Walley, John Marshall, Claudia C dos Santos, Brent W Winston, Asher A Mendelson, Chintan Dave, Lauralyn McIntyre

**Affiliations:** Clinical Epidemiology Program, The Ottawa Hospital Research Institute, Ottawa, ON, Canada; Department of Medicine, University of British Columbia, Vancouver, BC, Canada; Regenerative Medicine Program, The Ottawa Hospital Research Institute, Ottawa, ON, Canada; Clinical Epidemiology Program, The Ottawa Hospital Research Institute, Ottawa, ON, Canada; Clinical Epidemiology Program, The Ottawa Hospital Research Institute, Ottawa, ON, Canada; Department of Medicine, University of Ottawa, Ottawa, ON, Canada; Clinical Epidemiology Program, The Ottawa Hospital Research Institute, Ottawa, ON, Canada; Regenerative Medicine Program, The Ottawa Hospital Research Institute, Ottawa, ON, Canada; Department of Cell and Molecular Medicine, University of Ottawa, Ottawa, ON, Canada; Department of Anesthesiology and Pain Medicine, University of Ottawa, The Ottawa Hospital, Ottawa, ON, Canada; Regenerative Medicine Program, The Ottawa Hospital Research Institute, Ottawa, ON, Canada; Department of Cell and Molecular Medicine, University of Ottawa, Ottawa, ON, Canada; Department of Medicine, Centre for Heart Lung Innovation, University of British Columbia, Vancouver, BC, Canada; Department of Surgery (Critical Care), University of Toronto, Toronto, ON, Canada; Keenan Research Centre for Biomedical Science and Interdepartmental Division of Critical Care, St. Michael’s Hospital, University of Toronto, Toronto, ON, Canada; Department of Critical Care Medicine, University of Calgary, Calgary, AB, Canada; Section of Critical Care Medicine, Department of Medicine, Rady Faculty of Health Sciences, University of Manitoba, Winnipeg, MB, Canada; Division of Critical Care Medicine, Department of Medicine, Western University, London, ON, Canada; Clinical Epidemiology Program, The Ottawa Hospital Research Institute, Ottawa, ON, Canada; Department of Medicine (Division of Critical Care), University of Ottawa, Ottawa, ON, Canada; Department of Medicine (Critical Care), The Ottawa Hospital, Ottawa Hospital Research Institute, Centre for Transfusion and Critical Care Research, Ottawa, ON, Canada

**Keywords:** mesenchymal stromal (stem) cell, umbilical cord, sepsis, preclinical, systematic review

## Abstract

**Background:**

In preclinical studies, mesenchymal stromal cells (MSCs), including umbilical cord-derived MSCs (UC-MSCs), demonstrate the ability to modulate numerous pathophysiological processes related to sepsis; however, a systematic synthesis of the literature is needed to assess the efficacy of UC-MSCs for treating sepsis.

**Objective:**

To examine the effects of UC-MSCs on overall mortality (primary outcome) as well as on organ dysfunction, coagulopathy, endothelial permeability, pathogen clearance, and systemic inflammation (secondary outcomes) at prespecified time intervals in preclinical models of sepsis.

**Methods:**

A systematic search was conducted on Embase, Ovid MEDLINE, and Web of Science up to June 20, 2023. Preclinical controlled studies using in vivo sepsis models with systemic UC-MSC administration were included. Meta-analyses were conducted and expressed as odds ratios (OR) and ratios of the weighted means with 95% CI for categorical and continuous data, respectively. Risk of bias was assessed with the SYRCLE tool.

**Results:**

Twenty-six studies (34 experiments, *n* = 1258 animals) were included in this review. Overall mortality was significantly reduced with UC-MSC treatment as compared to controls (OR: 0.26, 95% CI: 0.18-0.36). At various prespecified time intervals, UC-MSCs reduced surrogate measures of organ dysfunction related to the kidney, liver, and lung; reduced coagulopathy and endothelial permeability; and enhanced pathogen clearance from multiple sites. UC-MSCs also modulated systemic inflammatory mediators. No studies were rated as low risk across all SYCLE domains.

**Conclusions:**

These results demonstrate the efficacy of UC-MSC treatment in preclinical sepsis models and highlight their potential as a therapeutic intervention for septic shock.

## Graphical Abstract

**Graphical Abstract:**
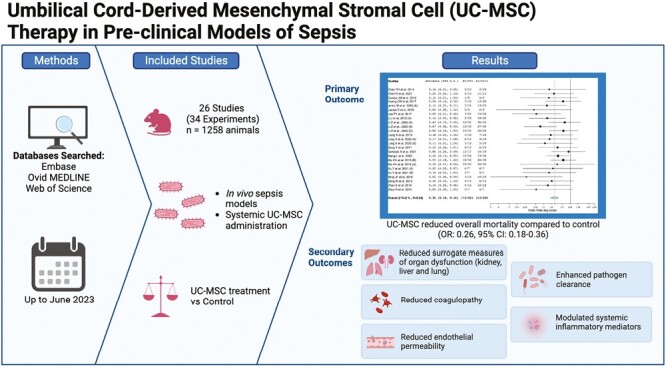

**Significance Statement**

Mesenchymal stromal cells (MSCs) have emerged as potential therapeutics for the treatment of complex life-threatening conditions such as sepsis. From our preclinical sepsis meta-analyses, umbilical cord-derived MSCs as compared to controls reduced death, surrogate measures of organ dysfunction, inflammation and endothelial permeability, and enhanced pathogen clearance, thereby providing the rationale and justification for studying them in the clinical setting of sepsis and septic shock.

## Introduction

Sepsis is defined as a syndrome of life-threatening organ dysfunction caused by a dysregulated host response to infection.^1^ Underlying biological changes that underpin features of sepsis, which may be amenable to treatment (ie, “treatable traits”), are often the focus of emerging therapeutics directed toward the excessive production of inflammatory cytokines, endothelial permeability, coagulopathies, organ failures, and death.^2-5^ Despite decades of research focused on immunotherapies and modulation of underlying biological drivers of disease, supportive care remains the mainstay of therapy for sepsis.^6,7^ As septic shock is associated with a high mortality rate of 20%-40%, identifying effective therapeutics remains an important area of research.^8,9^

Mesenchymal stromal cells (MSCs) are multipotent stem cells found in tissues such as bone marrow, adipose tissue, and umbilical cord.^10^ Recent preclinical sepsis studies suggest that MSCs represent a potential novel therapeutic for the treatment of sepsis. MSCs have been found to modulate the inflammatory response,^3,11,12^ mitigate coagulopathy, and increase endothelial permeability largely through paracrine actions.^13,14^ MSCs have also demonstrated the ability to enhance pathogen clearance, reduce death, and improve surrogate measures of organ failure in preclinical studies.^15,16^

While all MSCs share similar biological characteristics, such as the expression of certain markers (eg, CD105, CD90, and CD73) and differentiation capabilities into osteoblasts, adipocytes, and chondroblasts, in vitro studies suggest they may vary in other capacities by having distinct gene expression profiles and immunomodulatory activities.^17-19^ Despite these in vitro differences, systematic reviews of animal models of sepsis that compared the in vivo efficacy of bone marrow-derived MSCs (BM-MSCs)^20^ and all MSC types^21,22^ to controls demonstrated reductions in death. Furthermore, subgroup analyses from the latter reviews suggested similar reductions in mortality for different MSC types (ie, adipose MSCs, BM-MSCs, umbilical cord-derived MSCs [UC-MSCs], IL-10-enhanced UC-MSCs, and menstrual fluid MSCs) as compared to controls.^21,22^ Although the reductions in death in animal models of sepsis appear similar for different MSC types, UC-MSCs have important advantages which include ready availability from donors, plentifulness, ease of procurement, and a noninvasive procedure to harvest.^23,24^ UC-MSCs also have the added advantage of being derived from very young tissue, with greater proliferative potential compared to cells from adult sources, making them a viable “off-the-shelf” product and a potentially less expensive therapeutic for sepsis.^24^ Due to these advantages, our team is planning a phase II randomized controlled trial (RCT) comparing UC-MSCs to placebo for septic shock (Umbilical cord cellular immunotherapy for septic shock: a phase II RCT (UC-CISS II), NCT05969275).

In preparation for the UC-CISS II RCT, our team evaluated the effects of UC-MSCs, as compared to controls, in preclinical models of sepsis as no previous systematic review has reported exclusively on UC-MSCs. Furthermore, no systematic review has summarized the effects of UC-MSCs on surrogate measures of organ dysfunction, or other central aspects of sepsis pathobiology including coagulopathy, endothelial permeability, pathogen clearance, and systemic inflammation in preclinical models of sepsis. The evidence generated from this systematic review will provide hypotheses related to the biological effects of UC-MSCs, as well as the consideration of potential outcome measures for the UC-CISS II RCT and for other UC-MSC trials in sepsis going forward into the future.

## Materials and Methods

The protocol for this review was posted on the University of Ottawa’s Open Access Research Institutional Repository (http://hdl.handle.net/10393/43645). The approach for this systematic review and meta-analysis was adapted from a previously published systematic review by our group.^21^

### Eligibility Criteria

We included all preclinical controlled studies that used in vivo models of systemic sepsis, as mimicked through systemic infection (eg, cecal ligation and puncture [CLP], bacteria administration, and fecal slurry administration) or systemic endotoxin administration (eg, lipopolysaccharides [LPS] alone or with d-galactosamine [D-gal]), along with the systemic administration of UC-MSCs within 24 hours after sepsis induction. We excluded studies that used neonatal animal models of sepsis and models of direct acute lung injury. Studies that used engineered or modified MSCs (eg, altered the expression of a particular gene), multiple doses of MSCs, and co-treatments with other therapies (excluding antibiotics) or cell types were also excluded.

### Intervention and Control Groups

The intervention group is defined as animals treated with UC-MSCs systemically following the induction of sepsis. The comparator or control group is defined as animals treated with vehicle or other controls (eg, phosphate-buffered saline, normal saline, fibroblasts, hydroxyethyl starch, Dulbecco’s Modified Eagle Medium, and no treatment) following the induction of sepsis.

### Literature Search

We conducted electronic literature searches on Embase, Ovid MEDLINE, and Web of Science using search strategies developed in conjunction with an information specialist to identify relevant studies published up to June 20, 2023. The full search strategy can be found in Supplementary Appendix. Additional searches were conducted by reviewing the bibliographies of eligible studies, reviews, abstracts, and relevant conference proceedings.

### Study Screening and Selection

All studies were independently screened by 2 reviewers (CH and UT) on Covidence to determine if they met the inclusion requirements. Discrepancies were resolved through discussion with a third team member (LM).

### Primary Outcome

The primary outcome was overall mortality measured at the latest time point reported. The overall mortality results were further analyzed according to subgroups determined a priori. These included animal species (eg, mice, rat, pig, and immunocompromised mice), sepsis model (eg, CLP, LPS, LPS with D-gal, live bacteria, and fecal slurry injection), resuscitation (eg, fluid alone, antibiotics, fluid and antibiotics, and no resuscitation), dose of MSC (eg, ≤500 000 cells, 500 000 to 1 × 10^6^ cells, >1 × 10^6^ cells), timing of MSC administration (eg, ≤1 hour, 1-6 hours, ≥6 hours [up to 24 hours] post sepsis induction), MSC preparation (eg, fresh or frozen), and the MSC administration route (eg, intravenous or intraperitoneal). Mortality outcomes were also measured for a priori determined time intervals of within 2 days, 2-4 days, and greater than 4 days, as per a previous review.^21^

### Secondary Outcomes

The secondary outcomes included surrogate measures of organ dysfunction, coagulopathy, endothelial permeability, pathogen clearance, and systemic inflammation, as these are cardinal features of the pathobiology of sepsis.^5^ Surrogate measures of organ dysfunction relating to the kidney (eg, creatinine and blood urea nitrogen [BUN]), liver (eg, alanine transaminase [ALT] and aspartate aminotransferase [AST]), lung (eg, neutrophils and myeloperoxidase activity [MPO]), and heart (eg, echocardiographic assessment of function such as ejection fraction and fractional shortening, arterial lactate) were measured at 0-6, 7-24, 25-48, 49-72, and >72 hours post sepsis induction. Outcome measures for coagulopathy (eg, platelets, fibrinogen, pro-thrombin time [PT], and activated partial thromboplastin time [APTT]), endothelial permeability (eg, bronchoalveolar lavage [BAL] protein, BAL albumin, and lung wet/dry ratio), pathogen clearance (eg, in the blood, peritoneum, kidney, spleen, liver, lung, and peritoneal lavage fluid [PLF]), and circulating or systemic inflammatory markers in the plasma (eg, TNFα, IL-1β, IFN-ɣ, IL-6, IL-10, IL-8 or equivalent homologs, MCP-1 or equivalent homologs) were also measured at 0-6, 7-24, 25-48, 49-72, and >72 hours post sepsis induction. When an experiment measured an outcome more than once in one of our prespecified time intervals, the outcome at the latest time point was used for analysis.

### Data Extraction

Information on the animal model (eg, species, strain, age, weight, and sex), sepsis model, sample size, resuscitation, MSC characteristics (eg, origin, source, condition, and dose), control group, timing of UC-MSC delivery and administration route, along with the primary and secondary outcomes of interest, were extracted by 2 independent reviewers (CH and UT) using a predesigned standardized template. An experiment must have an intervention group and a corresponding control group for comparison. All data presented in graphical format were extracted using ImageJ (https://imagej.nih.gov/ij/). Discrepancies in the extracted data were resolved by a third team member (LM). Authors were contacted when further clarification of the data was required.

### Risk of Bias Assessment

The SYRCLE risk of bias (ROB) tool for preclinical animal studies was used to evaluate the methodological quality of the included studies and assess elements for “high,” “low,” or “unclear” risk.^25^ Specific domains were identified a priori and evaluated independently by 2 reviewers (CH and CD) with discrepancies resolved through discussion with a third team member (LM). The 10 domains evaluated include (1) sequence generation, (2) baseline characteristics, (3) allocation concealment, (4) random housing, (5) blinding of personnel, (6) random outcome assessment, (7) blinding of outcome assessors, (8) completeness of outcome data, (9) selective outcome reporting, and (10) other sources of bias (eg, conflicts of interest, funding sources, a priori sample size calculations, etc.).

### Statistical Analysis

Mortality results were pooled using a random effects model (Comprehensive Meta-Analysis Version 3.3070) to express results as odds ratios (OR) and 95% CI. Results from outcomes with continuous data (secondary outcomes) were pooled using a random effects model; these outcomes were expressed as the ratio of the weighted means (ROM) and 95% CI. A random effects model was used because it takes into account both within-study and between-study variation and the CI around the point estimate is often wider compared to if a fixed-effects model were used.^26^ An OR or ROM of <1 favored the UC-MSC group as compared to the control group. To reduce measurement bias and in accordance with our protocol, time of sepsis induction was the anchor used for the description of all outcome measures in accordance with the prespecified time intervals. Heterogeneity of the results was also assessed using the *I*^2^ statistic and interpreted according to commonly adopted thresholds derived from Cochrane systematic review methodologies: 0%-40% may not be important, 30%-60% moderate heterogeneity, 50%-90% substantial heterogeneity, and 75%-100% considerable heterogeneity.^27^ The presence of publication bias was assessed through visual inspection of a funnel plot. Subsequent trim and fill analysis was used to estimate the number of missing studies and provide an estimated UC-MSC effect size “adjusted” for the publication bias.^28^

## Results

### Search Results and Study Characteristics

**Figure 1.**
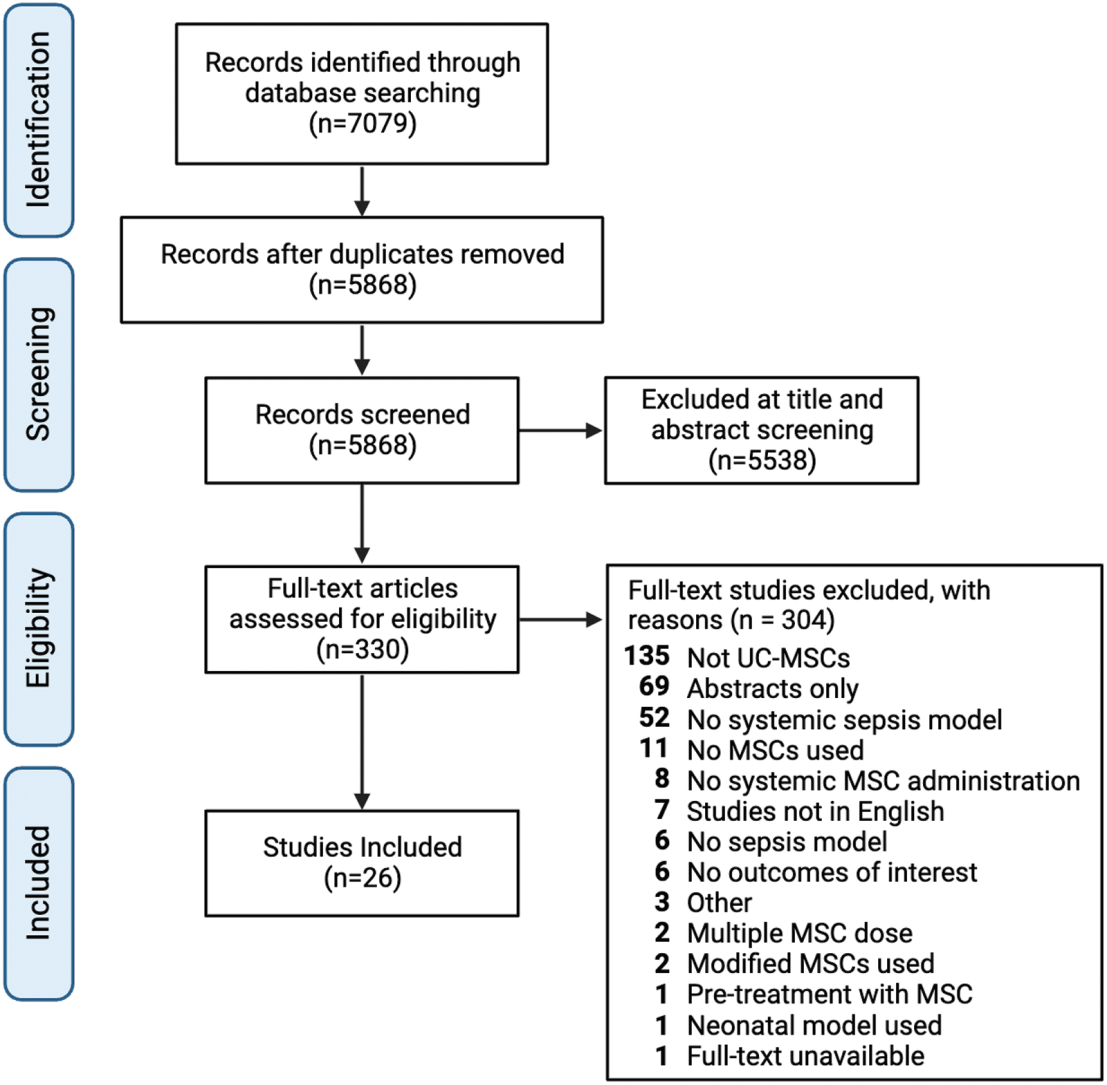
Preferred reporting items for systematic reviews and meta-analysis flow diagram for literature search and study inclusion.

Our systematic search strategy yielded 7079 potential studies. After removing duplicates and applying our eligibility criteria, 26 studies comprised of 34 experiments (*n* = 1258 animals) were included in this review^29-54^ (Fig. 1). Complete reporting of the included studies can be found in Table 1. A summary of the study characteristics (including the animal type, sepsis model, resuscitation, MSC timing, MSC preparation, MSC dose, and MSC administration route) can be found in Supplementary Table S1.

**Table 1.** Summary of the included preclinical studies.

Author ^i^, year	Animal model (species, strain)	Age (weeks), weight (g), sex	Sepsis model	Sample size^a^	Resuscitation	UC-MSC intervention group				Control group	Time of delivery (hours post sepsis induction)	Administration route
						Origin^b^	Source	Condition (fresh^c^ or frozen)	Dose			
Capcha et al., 2019^29^	Rat, Wistar	NR, 200-300, M	CLP (2 × 16G)	14	F, Anti	UC	Xeno	Fresh	1.0 × 10^6^	Saline	6	IP
Chao et al., 2014^30^	Rat, Wistar	NR, 250-300, M	CLP (1 × 18G)	18 Survival studies: 20	NR	BM, UC	Xeno	Fresh	5.0 × 10^6^	PBS	4	IV
Chen et al., 2019^31^	Rat, Sprague-Dawley	NR, NR, M	LPS + D-gal (i.p.)	10	NR	UC	Xeno	Fresh	1.0 × 10^7^	Saline	2	IV
Chen J et al., 2021^32^	Mouse, C57BL/6	8-10, NR, M	CLP (2 × 21G)	12	F	UC	Xeno	Unclear^d^	1.0 × 10^6^	Saline	6	IV
Chen R et al., 2021^33^	Rat, Sprague-Dawley	8-10, NR, M	CLP (3 × 21G)	12 Survival studies: 25	N	UC, AF	Xeno	Fresh	1.0 × 10^6^	Saline	6	IV
Condor et al., 2016^34^	Rat, Wistar	NR, 200-280, M	CLP (2 × 16G)	16 Survival studies: 18	F, Anti	UC	Xeno	Fresh	1.0 × 10^6^	Saline	6	IP
Huang et al., 2017^35^^e^	Rat, Sprague-Dawley	8, 180-220, M	LPS (i.p.)	15 Survival studies: 30	NR	UC	Xeno	Fresh	5.0 × 10^5^	Saline and fibroblast	1	IV
Jerkic et al., 2020 A^36^	Rat, Sprague-Dawley	NR, 350-450, M	Fecal slurry	26 Survival studies: 41	F	UC	Xeno	Fresh	1.0 × 10^7^	PBS	4	IV
Jerkic et al., 2020 B^36^^f^	Rat, Sprague-Dawley	NR, 350-450, M	Fecal slurry	37	F	UC	Xeno	Fresh	2.0 × 10^6^, 5.0 × 10^6^, 1.0 × 10^7^	PBS	4	IV
Laroye et al., 2018^37^	Pig, NR	26, 40000-60000, M	Fecal slurry	12 Survival studies: 12 (same animals)	F	UC	Xeno	Frozen	1.0 × 10^6^	HES	4	IV
Lee et al., 2017^38^	Rat, Sprague-Dawley	NR, 325-350, M	CLP (2 × 18G)	12 Survival studies: 50	NR	UC	Xeno	Frozen	1.2 × 10^6^	Saline	1	IV
Li et al., 2016^39^	Rat, Sprague-Dawley	NR, 240-280, F	LPS (i.p.)	6	NR	UC	Xeno	Fresh	5.0 × 10^5^	PBS	1	IV
Li et al., 2012 A^40^	Rat, Sprague-Dawley	NR, 240-280, M	LPS (i.p.)	Survival studies: 60	NR	UC	Xeno	Fresh	5.0 × 10^5^	Saline	1	IV
Li et al., 2012 B^40^	Rat, Sprague-Dawley	NR, 240-280, M	LPS (i.p.)	10	NR	UC	Xeno	Fresh	5.0 × 10^5^	Saline	1	IV
Li et al., 2020 A^41^^g^	Mouse, C57BL/6J	NR, 22-25, M	CLP (NR)	Survival studies: 60	F, Anti	UC	Xeno	Unclear^d^	2.0 × 10^5^	PBS	3	IV
Li et al., 2020 B^41^^h^	Mouse, C57BL/6J	NR, 22-25, M	CLP (NR)	Survival studies: 60	F, Anti	UC	Xeno	Unclear^d^	2.0 × 10^5^	PBS	3	IV
Li et al., 2020 C^41^	Mouse, C57BL/6J	NR, 22-25, M	*S. aureus* (1 × 10^9^ CFU, i.p.)	Survival studies: 60	F, Anti	UC	Xeno	Unclear^d^	2.0 × 10^5^	PBS	3	IV
Li et al., 2020 D^41^	Mouse, C57BL/6J	NR, 22-25, M	CLP (NR)	10-12	F	UC	Xeno	Unclear^d^	2.0 × 10^5^	PBS	3	IV
Liang et al., 2019^42^	Rat, Sprague-Dawley	NR, 180-220, M	CLP (1 × 18G)	12 Survival studies: 20	NR	UC	Xeno	Unclear^d^	2.5 × 10^6^	PBS	0	IV
Liu et al., 2021^43^	Mouse, C57BL/6L	NR, 20-22, M	LPS + D-gal (i.p.)	12	NR	UC	Xeno	Fresh	1.0 × 10^6^	NR	6	IV
Long et al., 2020 A^44^	Mouse, ICR	6, 28-32, M	*E. coli* (1 × 10^8^ CFU, i.p.)	10 Survival studies: 20	NR	UC	Xeno	Fresh	1.0 × 10^6^	PBS	4	IV
Long et al., 2020 B^44^	Mouse, ICR	6, 28-32, M	*E. coli* (1 × 10^8^ CFU, i.p.)	10 Survival studies: 20	Anti	UC	Xeno	Fresh	1.0 × 10^6^	PBS	4	IV
Song et al., 2017^45^	Mouse, C57BL/6	NR, 25-30, M	CLP (NR)	10-16 Survival studies: 22	F	UC	Xeno	Unclear^d^	1.0 × 10^6^	PBS	4	IV
Varkouhi et al., 2021^46^	Mouse, C57BL/6	10-11, 22-28, M	CLP (1 × 21G)	14 Survival studies: 36	F, Anti	BM, UC	Xeno	Fresh	25 × 10^4^	PBS	6	IV
Wang et al., 2019^47^	Rat, Sprague-Dawley	7-10, 300-350, NR	LPS + D-gal(i.p.)	12 Survival studies: NR	NR	UC	Xeno	Fresh	5.0 × 10^6^	Saline	1	IP
Wang et al., 2022^48^	Rat, Sprague-Dawley	91, 722-881, M	CLP (1 × 22G)	90 Survival studies: 72	NR	UC	Xeno	Fresh	5.0 × 10^6^	Saline	1	IV
Wu et al., 2016 A^49^	Mouse, C57BL/6	6, 25, M	CLP (1 × 21G)	14 Survival studies: 60	F	UC	Xeno	Fresh	1.0 × 10^6^	PBS	0	IP
Wu et al., 2016 B^49^	Mouse, C57BL/6	6, 25, M	CLP (1 × 21G)	NR Survival studies:60	F, Anti	UC	Xeno	Fresh	1.0 × 10^6^	PBS	0	IP
Xu et al., 2021 A^50^	Mouse, C57BL/6	NR, 25-30, M	CLP (NR)	14 Survival studies: 10-14	F	UC	Xeno	Unclear^d^	1.0 × 10^6^	PBS	4	IV
Xu et al., 2021 B^50^	Mouse, C57BL/6	NR, 25-30, M	LPS (i.p.)	14 Survival studies: 10-14	NR	UC	Xeno	Unclear^d^	1.0 × 10^6^	PBS	4	IV
Yang et al., 2015^51^	Mouse, NR	4-8, 20-25, M	LPS + D-gal (i.p.)	Survival studies: 20	NR	UC	Xeno	Fresh	5.0 × 10^5^	DMEM	0	IV
Zeng et al., 2015^52^	Mouse, NOD/SCID	6, 20, M	LPS + D-gal (i.p.)	24 Survival studies: 24	NR	UC	Xeno	Fresh	2.0 × 10^6^	PBS	6	IV
Zhao et al., 2014^53^	Mouse, C57BL/6	NR, 25-30, M	CLP (NR)	20-24 Survival studies: 24	NR	UC	Xeno	Fresh	1.0 × 10^6^	Saline	1	IV
Zhou et al., 2014^54^	Mouse, NOD/SCID	6, 20, M	LPS + D-gal (i.p.)	14 Survival studies: 14	NR	UC	Xeno	Fresh	2.0 × 10^6^	PBS	6	IV

^a^Sample size reflects the number of animals included in our analysis, not all the animals used in the study.

^b^Some studies used MSCs from multiple sources however we only report on the UC-MSCs in this study.

^c^UC-MSC preparation was described as fresh or enough detail was provided to assume freshly cultured.

^d^UC-MSC preparation was unclear and not enough detail was presented to assume fresh or frozen.

^e^This study had 2 control groups whose results were combined for analysis.

^f^This experiment had multiple UC-MSC groups whose results were combined for analysis.

^g^In this experiment, antibiotics were given 24 hours after sepsis induction.

^h^In this experiment, antibiotics were given 6 hours after sepsis induction. i Some studies were extracted as multiple experiments (as defined by having independent control and intervention groups for comparison) and were denoted with a letter (i.e., A, B, C, D) Abbreviations: UC-MSC, umbilical cord-derived mesenchymal stromal cells; NR, not reported; NOD/SCID, non-obese diabetic severe combined immune-deficient; CLP, cecal ligation and puncture; LPS, lipopolysaccharide; LPS + D-gal, lipopolysaccharide and d-galactosamine; N, none; F, fluids; Anti, antibiotics; UC, umbilical cord; BM, bone marrow; AF, amniotic fluid; Xeno, xenogeneic; PBS, phosphate-buffered saline; HES, hydroxyethyl starch; DMEM, Dulbecco’s Modified Eagle Medium; IV, intravenous; IP, intraperitoneal.

### Primary Outcome: Effects of UC-MSCs on Mortality

**Figure 2.**
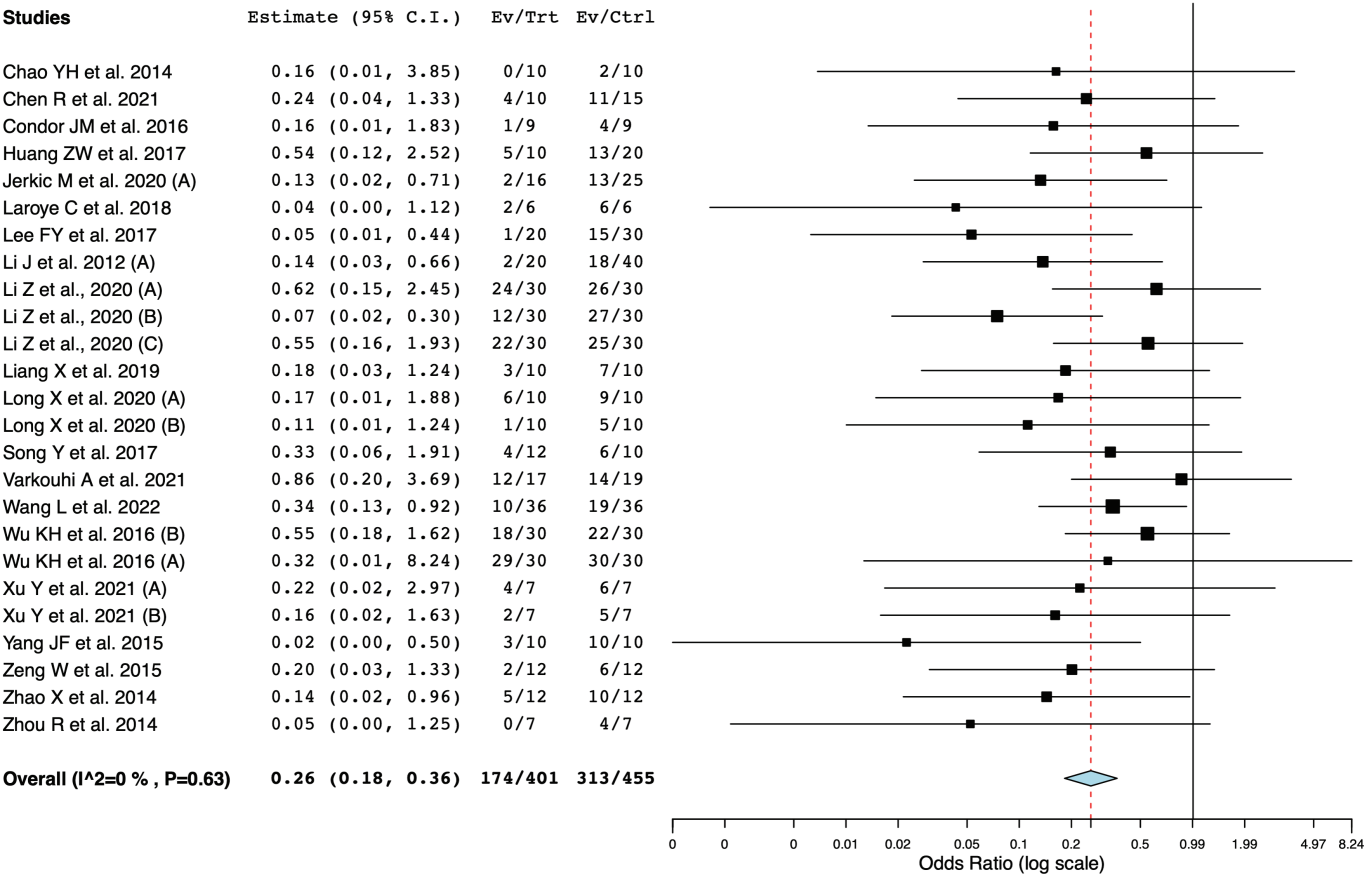
Forest plot summarizing the effects of UC-MSC treatment on overall mortality in preclinical models of sepsis. Point estimates represent the OR for each individual study with the size of the point depicting the relative contribution to the pooled effect. The corresponding horizontal line represents the 95% CI. Meta-analysis was performed on the pooled results with random effects modeling and the overall effect is depicted with the diamond.

From the 26 studies (34 experiments) included in this systematic review, 20 studies (25 experiments, *n* = 856) reported on mortality.^30,33-38,40-42,44-46,48-54^ UC-MSCs as compared to controls significantly reduced overall mortality with an OR of 0.26 and 95% CI of 0.18-0.36 (Fig. 2). Mortality was similarly reduced at all timepoints after sepsis induction: 2 days (OR: 0.27, 95% CI: 0.16-0.45),^30,33-38,40-42,44-46,48-54^ between 2 and 4 days (OR: 0.30, 95% CI: 0.20-0.45),^30,33-35,38,41,42,44-46,48-54^ and more than 4 days (OR: 0.25, 95% CI: 0.15-0.42)^30,34,35,38,41,42,45,46,50-52,54^ (Supplementary Fig. S1). Complete reporting of overall mortality results according to prespecified subgroups can be found in Table 2 and Supplementary Fig. S2. The administration of UC-MSCs as compared to controls was associated with a statistically significant reduction in overall mortality for all subgroups except the following: when UC-MSCs were administered to pigs (OR: 0.04, 95% CI: 0.00-1.12), no resuscitation measures were taken (OR: 0.24, 95% CI: 0.04-1.34), only antibiotic resuscitation was reported (OR: 0.11, 95% CI: 0.01-1.24), when ≤500 000 UC-MSCs were administered to rats (OR: 0.27, 95% CI: 0.07-1.06), and when MSCs were administered intraperitoneally (OR: 0.43, 95% CI: 0.17-1.12; Table 2; Supplementary Fig. S2).

**Table 2.** Overall mortality estimates of UC-MSC treatment according to prespecified subgroups.

Characteristic	Number of experiments	Mortality in control group (%)	Mortality in UC-MSC group (%)	Summary OR (95% CI)
*Animal*				
Mice	12	185/225 (82)	139/225 (62)	0.33 (0.21-0.54)^*^
Rat	9	102/195 (52)	28/141 (20)	0.22 (0.13-0.39)^*^
Pig	1	6/6 (100)	2/6 (33)	0.04 (0.00-1.12)
Immunocompromised mice	3	20/29 (69)	5/29 (17)	0.09 (0.02-0.4)^*^
*Sepsis model*				
CLP	14	199/278 (72)	127/263 (48)	0.29 (0.19-0.45)^*^
LPS	3	36/67 (54)	9/37 (24)	0.25 (0.09-0.68)^*^
LPS and D-gal	3	20/29 (69)	5/29 (17)	0.09 (0.02-0.40)^*^
Live bacteria	3	39/50 (78)	29/50 (58)	0.34 (0.12-0.93)^*^
Fecal slurry injection	2	19/31 (61)	4/22 (18)	0.10 (0.02-0.46)^*^
*Resuscitation*				
Fluid alone	5	55/63 (87)	42/65 (65)	0.21 (0.08-0.60)^*^
Antibiotics	1	5/10 (50)	1/10 (10)	0.11 (0.01-1.24)
Fluid + antibiotics	7	131/173 (76)	91/162 (56)	0.34 (0.17-0.68)^*^
None	1	11/15 (73)	4/10 (40)	0.24 (0.04-1.34)
Not reported	11	111/194 (57)	36/154 (23)	0.20 (0.11-0.35)^*^
*Dose of MSC (mouse)*				
≤500 000 cells	5	102/119 (86)	73/117 (62)	0.29 (0.10-0.90)^*^
500 000 to 1 × 10^6^ cells	8	93/116 (80)	69/118 (58)	0.29 (0.15-0.56)^*^
>1 × 10^6^ cells	2	10/19 (53)	2/19 (11)	0.14 (0.03-0.72)^*^
*Dose of MSC (rat)*				
≤500 000 cells	2	31/60 (52)	7/30 (23)	0.27 (0.07-1.06)
500 000 to 1 × 10^6^ cells	2	15/24 (63)	5/19 (26)	0.21 (0.05-0.85)^*^
>1 × 10^6^ cells	5	56/111 (50)	16/92 (17)	0.21 (0.10-0.42)^*^
*MSC timing*				
≤1 hour post sepsis induction	9	144/218 (66)	76/178 (43)	0.26 (0.15-0.45)^*^
1-6 hours post sepsis induction	11	130/175 (74)	79/168 (47)	0.23 (0.13-0.40)^*^
≥6 hours (up to 24 hours) post sepsis induction	5	39/62 (63)	19/55 (35)	0.31 (0.13-0.72)^*^
*MSC preparation*				
Fresh	16	190/295 (64)	100/249 (40)	0.27 (0.18-0.42)^*^
Frozen	2	21/36 (58)	3/26 (12)	0.05 (0.01-0.30)^*^
Unclear	7	102/124 (82)	71/126 (56)	0.28 (0.15-0.52)^*^
*MSC administration route*				
Intravenous	22	257/386 (67)	126/332 (38)	0.24 (0.16-0.35)^*^
Intraperitoneal	3	56/69 (81)	48/69 (70)	0.43 (0.17-1.12)

Abbreviations: MSC, mesenchymal stromal cells; UC-MSC, umbilical cord-derived MSCs; OR, odds ratio; CI, confidence interval; CLP, cecal ligation and puncture; LPS, lipopolysaccharide; D-gal, d-galactosamine.

^*^ *P* < 0.05.

### Secondary Outcomes

#### Effects of UC-MSCs on Surrogate Measures of Organ Dysfunction

##### Renal

Renal dysfunction was measured by creatinine concentrations (at 0-6,^37,48^ 7-24,^33,37,48^ 25-48,^41^ and 49-72 hours^48^ post sepsis induction) and BUN concentrations (at 0-6,^48^ 7-24,^33,48^ and 49-72 hours^48^ post sepsis induction; Table 3; Supplementary Fig. S3). Treatment with UC-MSCs, as compared to controls, was associated with a statistically significant reduction in creatinine at 49-72 hours post sepsis induction and BUN at 0-6 and 49-72 hours post sepsis induction.

**Table 3.** Secondary outcome estimates of UC-MSC treatment in preclinical models of sepsis.

Outcome	0-6 hours		7-24 hours		25-48 hours		49-72 hours		>72 hours	
	E (*n* control, n MSC)	ROM (95% CI)	E (*n* control, n MSC)	ROM (95% CI)	E (*n* control, n MSC)	ROM (95% CI)	E (*n* control, n MSC)	ROM (95% CI)	E (*n* control, n MSC)	ROM (95% CI)
*Renal*										
Creatinine	2 (11, 11)	0.95 (0.88-1.03)	3 (17, 17)	0.65 (0.29-1.50)	1 (5, 5)	0.61 (0.26-1.41)	1 (5, 5)	0.63 (0.58-0.68)^*^	—	—
BUN	1 (5, 5)	0.75 (0.65-0.86)^*^	2 (11, 11)	0.58 (0.24-1.42)	—	—	1 (5, 5)	0.68 (0.62-0.74)^*^	—	—
*Liver*										
ALT	2 (12, 12)	0.75 (0.39-1.45)	10 (64, 64)	0.69 (0.60-0.79)^*^	5 (32, 32)	0.65 (0.55-0.77)^*^	2 (17, 17)	0.60 (0.37-0.95)^*^	4 (33, 33)	0.90 (0.80-1.02)
AST	2 (12, 12)	1.00 (0.93-1.09)	10 (64, 64)	0.58 (0.45-0.76)^*^	5 (32, 32)	0.66 (0.58-0.75)^*^	2 (17, 17)	0.58 (0.53-0.62)^*^	4 (33, 33)	0.93 (0.70-1.22)
*Pulmonary*										
Pulm neutrophils	3 (18, 13)	0.87 (0.83-0.91)^*^	4 (24, 19)	0.78 (0.68-0.90)^*^	3 (18, 13)	0.84 (0.75-0.94)^*^	—	—	1 (10, 5)	0.91 (0.83-0.99)^*^
Pulm MPO	2 (15, 10)	0.81 (0.81-0.81)^*^	3 (22, 17)	0.73 (0.40-1.34)	2 (15, 10)	0.76 (0.26-2.22)	—	—	1 (10, 5)	0.92 (0.78-1.08)
*Cardiac*										
Ejection fraction	—	—	1 (7, 7)	1.05 (0.94-1.17)	—	—	—	—	—	—
Arterial lactate	1 (6, 6)	N/A^a^	1 (6, 6)	0.55 (0.21-1.45)	—	—	—	—	—	—
*Coagulopathy*										
Platelets	1 (6, 6)	1.14 (0.87-1.48)	1 (6, 6)	1.16 (0.70-1.91)	—	—	—	—	—	—
Fibrinogen	—	—	1 (6, 6)	1.79 (1.55-2.07)	—	—	—	—	—	—
PT	—	—	1 (6, 6)	0.38 (0.35-0.41)^*^	—	—	—	—	—	—
APTT	—	—	1 (6, 6)	0.34 (0.31-0.36)^*^	—	—	—	—	—	—
*Endothelial permeability*										
BAL protein	2 (8, 8)	0.87 (0.78-0.96)^*^	3 (14, 14)	0.76 (0.66-0.86)^*^	2 (8, 8)	0.78 (0.70-0.87)^*^	—	—	—	—
BAL albumin	—	—	—	—	—	—	—	—	1 (6, 6)	0.62 (0.58-0.67)^*^
Wet/dry	3 (18, 13)	0.97 (0.91-1.04)	4 (24, 19)	0.86 (0.83-0.89)^*^	3 (18, 13)	0.92 (0.87-0.98)^*^	—	—	1 (10, 5)	0.95 (0.86-1.04)
*Pathogen clearance*										
CFU blood	—	—	4 (23, 23)	0.05 (0.00-245.86)	3 (22, 22)	0.45 (0.32-0.62)^*^	—	—	—	—
CFU peritoneum	—	—	2 (10, 10)	0.01 (0.00-1.01)	2 (17, 17)	0.27 (0.00-18.26)	—	—	—	—
CFU kidney	—	—	—	—	1 (5, 5)	0.63 (0.44-0.89)^*^	—	—	—	—
CFU spleen	—	—	1 (7, 7)	0.04 (0.04-0.04)^*^	—	—	2 (22, 41)	0.64 (0.31-1.32)	—-	—
CFU liver	—	—	—	—	1 (5, 5)	0.77 (0.63-0.93)^*^	2 (22, 41)	0.27 (0.14-0.52)^*^	—	—
CFU lung	—	—	1 (7, 7)	0.08 (0.08-0.08)^*^	1 (5, 5)	0.61 (0.49-0.77)^*^	—	—	—	—
CFU PLF	—	—	—	—	—	—	1 (12, 14)	0.98 (0.28-3.50)	—	—
*Systemic inflammation*										
TNF-α	5 (33, 28)	0.66 (0.50-0.86)^*^	11 (69, 64)	0.67 (0.56-0.80)^*^	5 (34, 29)	0.67 (0.59-0.76)^*^	2 (17, 17)	0.47 (0.36-0.60)^*^	4 (36, 31)	0.84 (0.61-1.16)
IL-1β	2 (10, 10)	0.55 (0.31-0.99)^*^	7 (42, 42)	0.66 (0.57-0.75)^*^	2 (10, 10)	0.72 (0.52-1.01)	1 (5, 5)	0.53 (0.48-0.59)^*^	—	—
IFN-γ	2 (12, 12)	0.36 (0.16-0.84)^*^	3 (18, 18)	0.76 (0.50-1.14)	—	—	1 (5, 5)	0.53 (0.47-0.59)^*^	—	—
IL-6	5 (33, 28)	0.52 (0.49-0.56)^*^	11 (69, 64)	0.54 (0.41-0.71)^*^	5 (34, 29)	0.26 (0.08-0.83)^*^	2 (17, 17)	0.34 (0.09-1.24)	4 (36, 31)	0.52 (0.30-0.91)^*^
MCP-1	1 (7, 7)	0.26 (0.21-0.33)^*^	2 (16, 16)	0.80 (0.68-0.93)^*^	—	—	—	—	—	—
IL-10	4 (27, 22)	1.66 (1.09-2.53)^*^	9 (58, 53)	1.58 (1.01-2.46)^*^	5 (34, 29)	1.26 (1.01-1.58)^*^	1 (5, 5)	8.54 (8.05-9.06)^*^	3 (24, 19)	1.09 (0.92-1.29)

^a^ROM could not be determined as no variance or CI was provided in the study. Abbreviations: MSC, mesenchymal stromal cells; ROM, ratio of means; CI, confidence interval; BUN, blood urea nitrogen; ALT, alanine transaminase; AST, aspartate aminotransferase; Pulm, pulmonary; MPO, myeloperoxidase activity; PT, pro-thrombin time; APTT, activated partial thromboplastin time; BAL, bronchoalveolar lavage; CFU, colony-forming unit; PLF, peritoneal lavage fluid.

^*^ *P* < 0.05.

##### Liver

Liver dysfunction was measured by ALT and AST concentrations (at 0-6,^48,54^ 7-24,^31,33,34,42-44,47,48,52^ 25-48,^41,47,50,54^ 49-72,^48,52^ and >72 hours^50,52,54^ post sepsis induction; Table 3; Supplementary Fig. S4). Statistically significant reductions in ALT and AST were observed with UC-MSC treatment as compared to controls at 7-24, 25-48, and 49-72 hours post sepsis induction.

##### Pulmonary

Pulmonary dysfunction was measured by pulmonary neutrophils (at 0-6,^35,39,40^ 7-24,^32,35,39,40^ 25-48,^35,39,40^ and > 72 hours^35^ post sepsis induction) and MPO activity (at 0-6,^35,40^ 7-24,^29,35,40^ 25-48,^35,40^ and > 72 hours^35^ post sepsis induction; Table 3; Supplementary Fig. S5). Statistically significant reductions of pulmonary neutrophils were observed with UC-MSC treatment as compared to controls at all time points. MPO activity levels were also significantly reduced at 0-6 hours post sepsis induction with UC-MSC treatment.

##### Cardiac

Cardiac dysfunction was evaluated through measures of ejection fraction (at 7-24 hours^29^ post sepsis induction) and arterial lactate (at 0-6^37^ and 7-24 hours^37^ post sepsis induction; Table 3; Supplementary Fig. S6). No statistically significant change in ejection fraction or arterial lactate was observed with UC-MSC treatment as compared to controls.

#### Effects of UC-MSCs on Coagulopathy

Coagulopathy markers included measures of platelet concentrations (at 0-6^37^ and 7-24 hours^37^ post sepsis induction), fibrinogen (at 7-24 hours^33,37^ post sepsis induction), PT (at 7-24 hours^33^ post sepsis induction), and APTT (at 7-24 hours^33^ post sepsis induction; Table 3; Supplementary Fig. S7). Treatment with UC-MSCs as compared to controls did not demonstrate a statistically significant increase in platelets or fibrinogen; however, both PT and APTT levels were significantly reduced at 7-24 hours post sepsis induction.

#### Effects of UC-MSCs on Endothelial Permeability

Endothelial permeability was measured by BAL fluid protein concentrations (at 0-6,^39,40^ 7-24,^32,39,40^ and 25-48 hours^39,40^ post sepsis induction), BAL fluid albumin concentrations (at >72 hours^38^ post sepsis induction), and lung wet/dry ratio (at 0-6,^35,39,40^ 7-24,^33,35,39,40^ 25-48,^35,39,40^ and >72 hours^35^ post sepsis induction; Table 3; Supplementary Fig. S8). Significant reductions in BAL protein and albumin were observed at all prespecified time intervals with UC-MSC treatment as compared to controls. Lung wet/dry ratio was also significantly reduced with UC-MSC treatment at 7-24 and 25-48 hours post sepsis induction.

#### Effects of UC-MSCs on Pathogen Clearance

Pathogen clearance was measured by bacterial colony-forming unit (CFU) concentrations measured from blood samples (at 7-24^37,44,46^ and 25-48 hours^41,45,53^ post sepsis induction), peritoneum (at 7-24^44^ and 25-48^45,53^ hours post sepsis induction), kidney (at 25-48 hours^41^ post sepsis induction), spleen (at 7-24^46^ and 49-72 hours^36^ post sepsis induction), liver (at 25-48^41^ and 49-72 hours^36^ post sepsis induction), lung (at 7-24^46^ and 25-48 hours^41^ post sepsis induction), and PLF (at 49-72 hours^36^ post sepsis induction; Table 3; Supplementary Fig. S9). Statistically significant reductions in CFU were observed in the blood at 25-48 hours post sepsis induction, kidney at 25-48 hours post sepsis induction, spleen at 7-24 hours post sepsis induction, liver at 25-48 and 49-72 hours post sepsis induction, and lung at 7-24 and 25-48 hours post sepsis induction with UC-MSC treatment.

#### Effects of UC-MSCs on Systemic Inflammation

Systemic inflammation was measured by concentrations of TNF-α (at 0-6,^35,37,40,48,49^ 7-24,^30,31,33,35,37,40,42,44,46,48^ 25-48,^35,40,41,50^ 49-72,^48,52^ and >72 hours^35,50,52^ post sepsis induction), IL-1β (at 0-6,^40,48^ 7-24,^30,33,40,44,46,48^ 25-48,^40,41^ and 49-72 hours^48^ post sepsis induction), IFN-γ (at 0-6,^48,49^ 7-24,^42,46,48^ and 49-72 hours^48^ post sepsis induction), IL-6 (at 0-6,^35,37,40,48,49^ 7-24,^30,31,33,35,37,40,42,44,46,48^ 25-48,^35,40,41,50^ 49-72,^48,52^ and >72 hours^35,50,52^ post sepsis induction), MCP-1 (at 0-6^49^ and 7-24 hours^30,46^ post sepsis induction), and IL-10 (at 0-6,^35,40,48,49^ 7-24,^30,33,35,40,42,44,46,48^ 25-48,^35,40,41,50^ 49-72,^48^ and >72 hours^35,50^ post sepsis induction; Table 3; Supplementary Fig. S10). UC-MSC treatment was associated with a statistically significant reduction in circulating pro-inflammatory mediators including TNF-α at 0-6, 7-24, 25-48, and 49-72 hours post sepsis induction; IL-1β at 0-6, 7-24, and 49-72 hours post sepsis induction; IFN-γ at 0-6 and 49-72 hours post sepsis induction; IL-6 at 0-6, 7-24, 25-48, and >72 hours post sepsis induction; and MCP-1 at 0-6 and 7-24 hours post sepsis induction. Anti-inflammatory IL-10 levels were significantly increased with UC-MSC treatment as compared to controls at 0-6, 7-24, 25-48, and 49-72 hours post sepsis induction.

None of the 26 studies included in our systematic review reported harm individually. There were also no significant signals for harm found in the pooled analyses for our primary and secondary outcomes.

### ROB Assessment

Of the 26 studies included in this review, none were rated as low risk for all 10 domains assessed (Table 4; Supplementary Table S2). Three domains, including sequence generation (randomization), allocation concealment, and random housing, were identified to have unclear ROB in all studies as insufficient detail was provided in the methods to classify the level of risk. Four studies demonstrated a high ROB in one domain^38,45,50,52^ while 3 studies demonstrated high risk in 2 domains^44,53,54^; these 7 studies were all assessed to have a high ROB for selective outcome reporting due to the mortality and/or relevant secondary outcome being only presented in the results and not prespecified in the methods. One study was found to have high ROB for incomplete outcome data as the sample values (*n*) were not consistent between methods and results for some of the secondary outcomes.^54^ One included study also reported a conflict of interest with one of the authors having served as an unpaid section editor of the journal in which the study was published.^44^

**Table 4.** ROB assessment of included preclinical studies using the SYRCLE tool.

SYRCLE	Selection bias						Performance bias		Detection bias			Attrition bias		Reporting bias		Other bias			
Author, year	Sequence generation	Baseline characteristics				Allocation concealment	Random housing	Blinding of participants and personnel	Random outcome ossessment	Blinding of outcome assessors		Incomplete outcome data		Selective outcome reporting		Conflict of interest	Source of funding	Sample size calculation	Free of other high bias risk
		Age	Weight	Sex	Species strain					Mortality	Subjective secondary outcomes	Mortality	Secondary outcomes	Mortality	Secondary outcomes				
Capcha et al., 2019	U	U	L	L	L	U	U	L	L	N/A	L	N/A	L	N/A	L	L	L	U	L
Chao et al., 2014	U	U	L	L	L	U	U	U	U	U	N/A	U	U	L	L	L	L	U	L
Chen et al., 2019	U	U	U	L	L	U	U	U	U	N/A	N/A	N/A	U	N/A	L	L	L	U	L
Chen J et al., 2021	U	L	U	L	L	U	U	U	U	N/A	U	N/A	L	N/A	L	L	L	U	L
Chen R et al., 2021	U	L	U	L	L	U	U	U	U	U	U	L	L	L	L	L	L	U	L
Condor et al., 2016	U	U	L	L	L	U	U	U	U	U	N/A	L	U	L	L	L	L	U	L
Huang et al., 2017	U	L	L	L	L	U	U	U	U	U	U	L	U	L	L	L	L	U	L
Jerkic et al., 2020	U	U	L	L	L	U	U	U	L	U	U	U	U	L	L	L	L	L	L
Laroye et al., 2018	U	L	L	L	L	U	U	L	L	L	L	L	L	L	L	L	L	U	L
Lee et al., 2017	U	U	L	L	L	U	U	U	U	L	N/A	U	L	L	H	L	L	U	L
Li et al., 2016	U	U	L	L	L	U	U	U	U	N/A	U	N/A	U	N/A	L	U	L	U	L
Li et al., 2012	U	U	L	L	L	U	U	U	U	U	L	U	U	L	L	L	L	U	L
Li et al., 2020	U	U	L	L	L	U	U	U	L	U	U	U	U	L	L	L	L	U	L
Liang et al., 2019	U	U	L	L	L	U	U	U	U	U	N/A	U	U	L	L	L	L	U	L
Liu et al., 2021	U	U	L	L	L	U	U	U	U	N/A	N/A	N/A	L	N/A	L	L	L	U	L
Long et al., 2020	U	L	L	L	L	U	U	U	U	U	L	L	U	L	H	H	L	U	L
Song et al., 2017	U	U	L	L	L	U	U	U	U	U	U	U	U	L	H	L	L	U	L
Varkouhi et al., 2021	U	L	L	L	L	U	U	U	U	U	U	U	U	L	L	L	L	L	L
Wang et al., 2019	U	L	L	L	L	U	U	U	U	U	N/A	U	U	L	L	L	L	U	L
Wang et al., 2022	U	L	L	L	L	U	U	U	U	U	N/A	U	U	L	L	L	U	U	L
Wu et al., 2016	U	L	L	L	L	U	U	U	U	U	N/A	U	U	L	L	L	L	U	L
Xu et al., 2021	U	U	L	L	L	U	U	U	U	U	N/A	U	U	H	L	L	L	U	L
Yang et al., 2015	U	L	L	L	U	U	U	U	U	L	N/A	L	L	L	L	L	L	U	L
Zeng et al., 2015	U	L	L	L	L	U	U	U	U	L	N/A	L	N/A	H	L	L	L	U	L
Zhao et al., 2014	U	U	L	L	L	U	U	U	U	U	U	U	U	H	H	L	L	U	L
Zhou et al., 2014	U	L	L	L	L	U	U	U	U	U	N/A	L	H	H	L	L	L	U	L

H, high risk; L, low risk; U, unclear risk; N/A, not applicable.

### Assessment for Publication Bias

**Figure 3.**
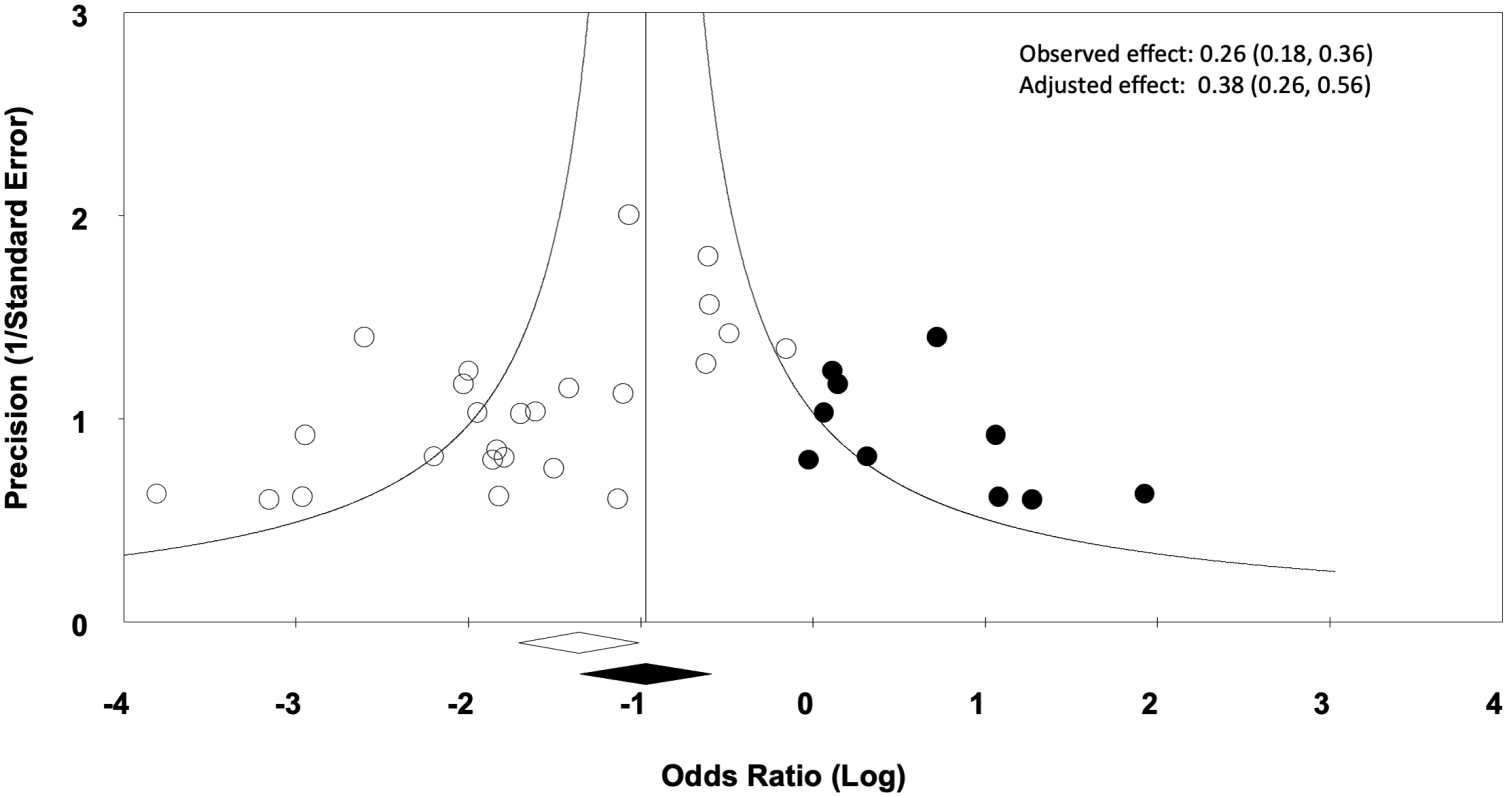
Funnel plot to detect publication bias. Trim and fill analysis was conducted on overall mortality results (the primary outcome). Open circles represent original study data and filled circles represent supplemented data through the trim and fill analysis. The open diamond represents the observed effect size and 95% CI. The filled diamond represents the adjusted effect size and 95% CI.

Visual inspection of the funnel plot analysis of the overall mortality data suggests that publication bias may exist due to a lack of small to medium studies reporting negative or null results (Fig. 3). In our analysis, UC-MSC treatment compared to controls demonstrated a reduction in overall mortality (OR: 0.26, 95% CI: 0.18-0.36); after the trim and fill analysis, the adjusted effect size (OR: 0.38, 95% CI: 0.26-0.56) was more modest but still significantly reduced.

## Discussion

Our comprehensive systematic review and meta-analysis summarizes the effects of UC-MSCs on overall mortality, surrogate measures of organ dysfunction, and key markers of sepsis pathobiology (coagulopathy, endothelial permeability, pathogen clearance, and systemic inflammation) in preclinical models of sepsis. Collectively, our review does suggest that UC-MSCs provide a benefit in preclinical sepsis without any significant risk for harm for any of the outcome measures. Our systematic review found that mortality was reduced in preclinical sepsis models with UC-MSC treatment, similar to what has been shown for all MSC types as reported by Lalu et al^21^ (OR: 0.27, 95% CI: 0.18-0.40) and Sun et al^22^ (OR: 0.29, 95% CI: 0.22-0.38). Prespecified subgroup analyses to examine the heterogeneity of the treatment effect found that UC-MSCs, as compared to controls, were associated with a reduction in overall mortality across different sepsis models (eg, CLP and LPS) and animal species (eg, mice and rats). In the one large animal (pig) model, mortality was lower in the MSC group as compared to the control group (2/6 [33%] vs 6/6 [100%]), but it did not reach statistical significance.^37^ Other potential sources of heterogeneity include the timing of MSC administration as well as the administration of fluid and antibiotic resuscitation. These co-interventions are part of the usual care that is delivered to humans with sepsis and are frequently omitted from preclinical models of sepsis.^55-57^ We observed that UC-MSC treatment reduced overall mortality even when MSC treatment was delayed to 6 hours post sepsis induction and when animals were resuscitated with fluids alone or in combination with antibiotics, thereby providing further evidence for the clinical applicability of UC-MSC therapy for sepsis. Additionally, overall mortality was significantly reduced for both freshly cultured and frozen UC-MSC preparations, supporting the use of frozen MSCs as an off-the-shelf product that would be necessary for acute treatment of time-sensitive syndromes such as sepsis.

The early stages of sepsis are characterized by the excessive production of pro-inflammatory markers, which contributes to the later hallmarks of organ dysfunction and damage.^5^ Our systematic review demonstrated that UC-MSCs improved surrogate measures of renal, liver, and pulmonary dysfunction; reduced coagulopathy and endothelial permeability; and enhanced pathogen clearance in at least one of our prespecified time intervals. Our review also suggests that UC-MSC therapy may modulate several sepsis inflammatory mediators with some of these changes sustained over time.^58^ The preclinical efficacy signals of our secondary outcome measures should be considered hypothesis generating as there was substantial statistical heterogeneity (*I*^2^ > 50)^59,60^ for some of the outcomes, which may be due to small sample sizes, as well as varying animal species, sepsis models, MSC doses, and time of MSC administration.

Our preclinical systematic review has several strengths. These include a systematic and transparent search for all available studies; a protocol that is accessible in a public repository and describes the design, methods, outcomes, and approach to the analyses; and an examination of the effect of UC-MSCs on our primary outcome according to subgroups to explore the heterogeneity of the treatment effect. Despite these strengths, our study also has limitations. While we attempted to include relevant sepsis-related biological mediators as secondary outcomes, due to immense sepsis complexity, there may be other important mediators that we did not examine. Our funnel plot analysis suggested the presence of publication bias. However, after adjustment using the trim and fill analysis method for hypothetical missing studies not published, overall mortality remained significantly reduced. The time of UC-MSC administration and measurement of the outcomes did vary in these studies, although all studies administered MSCs within the first 6 hours of sepsis induction. To reduce measurement bias and in accordance with our a priori protocol, we used sepsis induction as the anchor for the description of secondary outcomes at our prespecified time intervals. Due to the number of comparative tests performed, results from our subgroup analyses and secondary outcomes should be interpreted with caution and be considered hypothesis generating. None of the included studies were graded as low risk across all SYRCLE ROB domains. This was often due to a lack of clear or incomplete methodological reporting, which also limits the strength of the interpretation of our study findings. Finally, the utility of preclinical models of sepsis to predict clinical efficacy is often questioned because they do not entirely replicate the pathobiology of clinical sepsis, the human population with sepsis (eg, the use of small, young animals without comorbidities) and the clinical treatment setting (eg, time of MSC administration and the use of co-interventions such as fluids and antibiotics).^61^ The included studies in our review have similar limitations (ie, the inclusion of young animals with no morbidities) with the exception of the administration of co-interventions. In an a priori subgroup analysis, the effect of UC-MSCs on mortality was similar when fluids and antibiotics were administered compared to when neither of these co-interventions was administered. Furthermore, while preclinical animal sepsis studies do not fully reproduce the complexity and heterogeneity of clinical sepsis, they do provide hypotheses for potential UC-MSC effects that can then be tested in the setting of clinical trials.^62^

## Conclusion

The results of our systematic review suggested that UC-MSC therapy, as compared to controls, reduced mortality in preclinical models of sepsis. UC-MSCs also improved surrogate measures of organ dysfunction (in the kidney, liver, and lung), coagulopathy, endothelial permeability, pathogen clearance, and systemic inflammation in at least one of the prespecified time intervals. The evidence generated from this systematic review has assisted the planning of outcome measures for our UC-CISS II RCT and we hope for other UC-MSC trials in sepsis going forward into the future. Our review also provides hypotheses related to the potential biological effects of UC-MSCs in sepsis (eg, effect on coagulopathy, endothelial permeability, pathogen clearance, and inflammation) that investigators may want to consider for measurement in future UC-MSC clinical trials in sepsis.

## Supplementary Material

Supplementary material is available at *Stem Cells Translational Medicine* online.

szae003_suppl_Supplementary_Material

## Data Availability

The data underlying this article are available in the article and in its online supplementary material.

## References

[1] Singer M , Deutschman CS, Seymour C, et al. The third international consensus definitions for sepsis and septic shock (Sepsis-3). JAMA. 2016;315(8):801. 10.1001/JAMA.2016.0287 26903338 PMC4968574

[2] Nduka OO , Parrillo JE. The pathophysiology of septic shock. Crit Care Clin. 2009;25(4):677-702, vii. 10.1016/j.ccc.2009.08.002 19892247

[3] London NR , Zhu W, Bozza FA, et al. Targeting Robo4-dependent slit signaling to survive the cytokine storm in sepsis and influenza. Sci Transl Med. 2010;2(23):23ra19. 10.1126/scitranslmed.3000678 PMC2875996 20375003

[4] Maslove DM , Tang B, Shankar-Hari M, et al. Redefining critical illness. Nat Med. 2022;28(6):1141-1148. 10.1038/s41591-022-01843-x 35715504

[5] Hotchkiss RS , Moldawer LL, Opal SM, et al. Sepsis and septic shock. Nat Rev Dis Primers. 2016;2(1):1-21. 10.1038/nrdp.2016.45 PMC5538252 28117397

[6] Marshall JC. Why have clinical trials in sepsis failed? Trends Mol Med. 2014;20(4):195-203. 10.1016/j.molmed.2014.01.007 24581450

[7] Vincent JL , van der Poll T, Marshall JC. The end of “One Size Fits All” sepsis therapies: toward an individualized approach. Biomedicines. 2022;10(9):2260. 10.3390/biomedicines10092260 36140361 PMC9496597

[8] Kaukonen KM , Bailey M, Suzuki S, Pilcher D, Bellomo R. Mortality related to severe sepsis and septic shock among critically Ill patients in Australia and New Zealand, 2000-2012. JAMA. 2014;311(13):1308-1316. 10.1001/jama.2014.2637 24638143

[9] Rhee C , Klompas M. Sepsis trends: increasing incidence and decreasing mortality, or changing denominator? J Thorac Dis. 2020;12(Suppl 1):S89-S100. 10.21037/jtd.2019.12.51 32148931 PMC7024753

[10] Pittenger MF , Discher DE, Péault BM, et al. Mesenchymal stem cell perspective: cell biology to clinical progress. NPJ Regen Med. 2019;4(1):22. 10.1038/s41536-019-0083-6 31815001 PMC6889290

[11] Németh K , Leelahavanichkul A, Yuen PST, et al. Bone marrow stromal cells attenuate sepsis via prostaglandin E2–dependent reprogramming of host macrophages to increase their interleukin-10 production. Nat Med. 2009;15(1):42-49. 10.1038/nm.1905 19098906 PMC2706487

[12] Hoogduijn MJ , Popp F, Verbeek R, et al. The immunomodulatory properties of mesenchymal stem cells and their use for immunotherapy. Int Immunopharmacol. 2010;10(12):1496-1500. 10.1016/j.intimp.2010.06.019 20619384

[13] Fish KM , Hajjar RJ. Mesenchymal stem cells & endothelial function. EBioMedicine. 2015;2(5):376-377. 10.1016/j.ebiom.2015.04.015 26137582 PMC4485910

[14] Crisostomo PR , Wang M, Markel TA, et al. Stem cell mechanisms and paracrine effects. Shock. 2007;28(4):375-383. 10.1097/shk.0b013e318058a817 17577135

[15] Mei SHJ , Haitsma JJ, Dos Santos CC, et al. Mesenchymal stem cells reduce inflammation while enhancing bacterial clearance and improving survival in sepsis. Am J Respir Crit Care Med. 2010;182(8):1047-1057. 10.1164/rccm.201001-0010OC 20558630

[16] Gupta N , Krasnodembskaya A, Kapetanaki M, et al. Mesenchymal stem cells enhance survival and bacterial clearance in murine *Escherichia coli* pneumonia. Thorax. 2012;67(6):533-539. 10.1136/thoraxjnl-2011-201176 22250097 PMC3358432

[17] Hass R , Kasper C, Böhm S, Jacobs R. Different populations and sources of human mesenchymal stem cells (MSC): a comparison of adult and neonatal tissue-derived MSC. Cell Commun Signal. 2011;9(1):1-14. 10.1186/1478-811x-9-12 21569606 PMC3117820

[18] Strioga M , Viswanathan S, Darinskas A, Slaby O, Michalek J. Same or not the same? Comparison of adipose tissue-derived versus bone marrow-derived mesenchymal stem and stromal cells. Stem Cells Dev. 2012;21(14):2724-2752. 10.1089/scd.2011.0722 22468918

[19] Mattar P , Bieback K. Comparing the immunomodulatory properties of bone marrow, adipose tissue, and birth-associated tissue mesenchymal stromal cells. Front Immunol. 2015;6:560. 10.3389/fimmu.2015.00560 26579133 PMC4630659

[20] Ge L , Zhao J, Deng H, et al. Effect of bone marrow mesenchymal stromal cell therapies in rodent models of sepsis: a meta-analysis. Front Immunol. 2022;12:792098. 10.3389/fimmu.2021.792098 35046951 PMC8761857

[21] Lalu MM , Sullivan KJ, Mei SH, et al. Evaluating mesenchymal stem cell therapy for sepsis with preclinical meta-analyses prior to initiating a first-in-human trial. Elife. 2016;5:e17850. 10.7554/eLife.17850 27870924 PMC5153252

[22] Sun XY , Ding XF, Liang HY, Zhang XJ, Liu SH, Bing-Han, Duan XG, Sun TW. Efficacy of mesenchymal stem cell therapy for sepsis: a meta-analysis of preclinical studies. Stem Cell Res Ther. 2020;11(1):214. 10.1186/s13287-020-01730-7 32493435 PMC7268531

[23] Mebarki M , Abadie C, Larghero J, Cras A. Human umbilical cord-derived mesenchymal stem/stromal cells: a promising candidate for the development of advanced therapy medicinal products. Stem Cell Res Ther. 2021;12(1):1-10. 10.1186/S13287-021-02222-Y 33637125 PMC7907784

[24] Kern S , Eichler H, Stoeve J, et al. Comparative analysis of mesenchymal stem cells from bone marrow, umbilical cord blood, or adipose tissue. Stem Cells. 2006;24(5):1294-1301. 10.1634/STEMCELLS.2005-0342 16410387

[25] Hooijmans CR , Rovers MM, de Vries RB, et al. SYRCLE’s risk of bias tool for animal studies. BMC Med Res Methodol. 2014;14(1):43. 10.1186/1471-2288-14-43 24667063 PMC4230647

[26] Borenstein M , Hedges LV, Higgins JPT, Rothstein HR. A basic introduction to fixed-effect and random-effects models for meta-analysis. Res Synth Methods. 2010;1(2):97-111. 10.1002/jrsm.12 26061376

[27] Deeks JJ , Higgins JPT, Altman DG (editors). Chapter 10: Analysing data and undertaking meta-analyses. In: Higgins JPT, Thomas J, Chandler J, et al., eds. Cochrane Handbook for Systematic Reviews of Interventions version 6.4 (updated August 2023). *Cochrane*, 2023. https://training.cochrane.org/handbook/current/chapter-10

[28] Duval S , Tweedie R. Trim and fill: a simple funnel-plot–based method of testing and adjusting for publication bias in meta-analysis. Biometrics. 2000;56(2):455-463. 10.1111/j.0006-341x.2000.00455.x 10877304

[29] Capcha JMC , Rodrigues CE, Moreira R de S, et al. Wharton’s jelly-derived mesenchymal stem cells attenuate sepsis-induced organ injury partially via cholinergic anti-inflammatory pathway activation. Am J Physiol Regul Integr Comp Physiol. 2020;318(1):R135-R147. 10.1152/ajpregu.00098.2018 31596111

[30] Chao YH , Wu HP, Wu KH, et al. An increase in CD3+CD4+CD25 + regulatory T cells after administration of umbilical cord-derived mesenchymal stem cells during sepsis e110338. PLoS One. 2014;9(10):e110338. 10.1371/journal.pone.0110338 25337817 PMC4206342

[31] Chen H , Tang S, Liao J, Liu M, Lin Y. Therapeutic effect of human umbilical cord blood mesenchymal stem cells combined with G-CSF on rats with acute liver failure. Biochem Biophys Res Commun. 2019;517(4):670-676. 10.1016/j.bbrc.2019.07.101 31400854

[32] Chen J , Li C, Liang Z, et al. Human mesenchymal stromal cells small extracellular vesicles attenuate sepsis-induced acute lung injury in a mouse model: the role of oxidative stress and the mitogen-activated protein kinase/nuclear factor kappa B pathway. Cytotherapy. 2021;23(10):918-930. 10.1016/j.jcyt.2021.05.009 34272174

[33] Chen R , Xie Y, Zhong X, et al. MSCs derived from amniotic fluid and umbilical cord require different administration schemes and exert different curative effects on different tissues in rats with CLP-induced sepsis. Stem Cell Res Ther. 2021;12(1):1-12. 10.1186/s13287-021-02218-8 33676566 PMC7936453

[34] Cóndor JM , Rodrigues CE, de Sousa Moreira R, et al. Treatment with human Wharton’s Jelly-derived mesenchymal stem cells attenuates sepsis-induced kidney injury, liver injury, and endothelial dysfunction. Stem Cells Transl Med. 2016;5(8):1048-1057. 10.5966/sctm.2015-0138 27280799 PMC4954445

[35] Huang ZW , Liu N, Li D, et al. Angiopoietin-1 modified human umbilical cord mesenchymal stem cell therapy for endotoxin-induced acute lung injury in rats. Yonsei Med J. 2017;58(1):206-216. 10.3349/ymj.2017.58.1.206 27873515 PMC5122639

[36] Jerkic M , Gagnon S, Rabani R, et al. Human umbilical cord mesenchymal stromal cells attenuate systemic sepsis in part by enhancing peritoneal macrophage bacterial killing via heme oxygenase-1 induction in rats. Anesthesiology. 2020;132(1):140-154. 10.1097/ALN.0000000000003018 31764154

[37] Laroye C , Lemarié J, Boufenzer A, et al. Clinical-grade mesenchymal stem cells derived from umbilical cord improve septic shock in pigs. Intensive Care Med Exp. 2018;6(1):24. 10.1186/s40635-018-0194-1 30091119 PMC6082751

[38] Lee FY , Chen KH, Wallace CG, et al. Xenogeneic human umbilical cord-derived mesenchymal stem cells reduce mortality in rats with acute respiratory distress syndrome complicated by sepsis. Oncotarget. 2017;8(28):45626-45642. 10.18632/oncotarget.17320 28484089 PMC5542214

[39] Li D , Liu Q, Qi L, et al. Low levels of TGF-β1 enhance human umbilical cord-derived mesenchymal stem cell fibronectin production and extend survival time in a rat model of lipopolysaccharide-induced acute lung injury. Mol Med Rep. 2016;14(2):1681-1692. 10.3892/mmr.2016.5416 27357811

[40] Li J , Li D, Liu X, Tang S, Wei F. Human umbilical cord mesenchymal stem cells reduce systemic inflammation and attenuate LPS-induced acute lung injury in rats. J Inflamm. 2012;9(1):33. 10.1186/1476-9255-9-33 PMC3502090 22974286

[41] Li Z , Song Y, Yuan P, et al. Antibacterial fusion protein BPI21/LL-37 modification enhances the therapeutic efficacy of hUC-MSCs in sepsis. Mol Ther. 2020;28(8):1806-1817. 10.1016/j.ymthe.2020.05.014 32445625 PMC7403330

[42] Liang X , Li T, Zhou Q, et al. Mesenchymal stem cells attenuate sepsis-induced liver injury via inhibiting M1 polarization of Kupffer cells. Mol Cell Biochem. 2019;452(1-2):187-197. 10.1007/s11010-018-3424-7 30178273

[43] Liu M , He J, Zheng S, et al. Human umbilical cord mesenchymal stem cells ameliorate acute liver failure by inhibiting apoptosis, inflammation and pyroptosis. Ann Transl Med. 2021;9(21):1615-1615. 10.21037/atm-21-2885 34926659 PMC8640895

[44] Long X , Li X, Li T, et al. Umbilical cord mesenchymal stem cells enhance the therapeutic effect of imipenem by regulating myeloid-derived suppressor cells in septic mice. Ann Transl Med. 2021;9(5):404. 10.21037/atm-20-6371 33842625 PMC8033360

[45] Song Y , Dou H, Li X, et al. Exosomal miR-146a contributes to the enhanced therapeutic efficacy of interleukin-1β-primed mesenchymal stem cells against sepsis. Stem Cells. 2017;35(5):1208-1221. 10.1002/stem.2564 28090688

[46] Varkouhi AK , He X, Teixeira Monteiro AP, et al. Immunophenotypic characterization and therapeutics effects of human bone marrow- and umbilical cord-derived mesenchymal stromal cells in an experimental model of sepsis. Exp Cell Res. 2021;399(2):112473. 10.1016/j.yexcr.2021.112473 33428902

[47] Wang L , Li S, Wang HY, et al. In a rat model of acute liver failure, icaritin improved the therapeutic effect of mesenchymal stem cells by activation of the hepatocyte growth Factor/c-Met pathway. Evid Based Complement Alternat Med. 2019;2019:1-13. 10.1155/2019/4253846 PMC6935441 31915446

[48] Wang L , Deng Z, Sun Y, et al. The study on the regulation of Th Cells by mesenchymal stem cells through the JAK-STAT signaling pathway to protect naturally aged sepsis model rats. Front Immunol. 2022;13:820685. 10.3389/fimmu.2022.820685 35197984 PMC8858840

[49] Wu KH , Wu HP, Chao WR, et al. Time-series expression of toll-like receptor 4 signaling in septic mice treated with mesenchymal stem cells. Shock. 2016;45(6):634-640. 10.1097/SHK.0000000000000546 26682950

[50] Xu Y , Liu X, Li Y, et al. SPION-MSCs enhance therapeutic efficacy in sepsis by regulating MSC-expressed TRAF1-dependent macrophage polarization. Stem Cell Res Ther. 2021;12(1):1-20. 10.1186/s13287-021-02593-2 34627385 PMC8501658

[51] Yang JF , Cao HC, Pan QL, et al. Mesenchymal stem cells from the human umbilical cord ameliorate fulminant hepatic failure and increase survival in mice. Hepatobiliary Pancreat Dis Int. 2015;14(2):186-193. 10.1016/s1499-3872(15)60354-x 25865692

[52] Zeng W , Xiao J, Zheng G, et al. Antioxidant treatment enhances human mesenchymal stem cell anti-stress ability and therapeutic efficacy in an acute liver failure model. Sci Rep. 2015;5(1):11100. 10.1038/srep11100 26057841 PMC4460871

[53] Zhao X , Liu D, Gong W, et al. The toll-like receptor 3 Ligand, Poly(I:C), improves immunosuppressive function and therapeutic effect of mesenchymal stem cells on sepsis via inhibiting MiR-143. Stem Cells. 2014;32(2):521-533. 10.1002/stem.1543 24105952

[54] Zhou R , Li Z, He C, et al. Human umbilical cord mesenchymal stem cells and derived hepatocyte-like cells exhibit similar therapeutic effects on an acute liver failure mouse model. PLoS One. 2014;9(8):e104392. 10.1371/journal.pone.0104392 25101638 PMC4125182

[55] Manuel E , Ivers R, Guyen RN, et al. Early goal-directed therapy in the treatment of severe sepsis and septic shock. N Engl J Med. 2001;345(19):1368-1377. 10.1056/nejmoa010307 11794169

[56] Evans L , Rhodes A, Alhazzani W, et al. Surviving sepsis campaign: international guidelines for management of sepsis and septic shock 2021. Crit Care Med. 2021;49(11):e1063-e1143. 10.1097/CCM.0000000000005337 34605781

[57] Hellman J , Bahrami S, Boros M, et al. Part III: minimum quality threshold in preclinical sepsis studies (MQTiPSS) for fluid resuscitation and antimicrobial therapy endpoints. Shock. 2019;51(1):33-43. 10.1097/SHK.0000000000001209 29923896

[58] Jarczak D , Kluge S, Nierhaus A. Sepsis—pathophysiology and therapeutic concepts. Front Med. 2021;8:609. 10.3389/FMED.2021.628302 PMC8160230 34055825

[59] Higgins JPT , Thompson SG, Deeks JJ, Altman DG. Measuring inconsistency in meta-analyses. BMJ. 2003;327(7414):557-560. 10.1136/bmj.327.7414.557 12958120 PMC192859

[60] Friedrich JO , Adhikari NKJ, Beyene J. Ratio of means for analyzing continuous outcomes in meta-analysis performed as well as mean difference methods. J Clin Epidemiol. 2011;64(5):556-564. 10.1016/j.jclinepi.2010.09.016 21447428

[61] Dyson A , Singer M. Animal models of sepsis: why does preclinical efficacy fail to translate to the clinical setting? Crit Care Med. 2009;37(1 Suppl):S30-S37. 10.1097/CCM.0b013e3181922bd3 19104223

[62] Marshall JC , Deitch E, Moldawer LL, et al. Preclinical models of shock and sepsis: what can they tell us? Shock. 2005;24(Suppl 1):1-6. 10.1097/01.shk.0000191383.34066.4b 16374365

