# Supplementary material for: Efficacy and Safety of Umbilical Cord-Derived Mesenchymal Stromal Cell Therapy in Preclinical Models of Sepsis: A Systematic Review and Meta-analysis
Source: Stem Cells Transl Med. 2024 Feb 21;13(4):346–61. doi: 10.1093/stcltm/szae003 (PMC11016835; doi:10.1093/stcltm/szae003)
Supplement: szae003_suppl_Supplementary_Material [file szae003_suppl_supplementary_material.docx]

**Supplemental Table 1. Summary of study characteristics**

| **Characteristic** | **# Studies (%)** | **# Experiments (%)** |
| --- | --- | --- |
| **Total Included Studies/Experiments** | 26 | 34 |
| **Animal** |  |  |
| Rat | 13 (50) | 15 (44) |
| Mice | 9 (35) | 15 (44) |
| Immunocompromised Mice | 3 (12) | 3 (9) |
| Pig | 1 (4) | 1 (3) |
| **Sepsis Model^a^** |  |  |
| CLP | 14 (54) | 17 (50) |
| LPS | 4 (15) | 5 (15) |
| LPS and D-Gal | 6 (23) | 6 (18) |
| Live Bacteria | 2 (8) | 3 (9) |
| Fecal Slurry Injection | 2 (8) | 3 (9) |
| **Resuscitation^a^** |  |  |
| Fluid alone | 7 (27) | 8 (24) |
| Antibiotics | 1 (4) | 1 (3) |
| Fluid + antibiotics | 5 (19) | 7 (21) |
| Not Reported | 16 (62) | 17 (50) |
| None | 1 (4) | 1 (3) |
| **MSC Timing** |  |  |
| ≤1 hr post sepsis induction | 10 (38) | 12 (35) |
| 1 to 6 hrs post sepsis induction | 8 (31) | 14 (41) |
| ≥6 hrs (up to 24 hrs) post sepsis induction | 8 (31) | 8 (24) |
| **MSC Preparation** |  |  |
| Fresh | 19 (73) | 23 (68) |
| Frozen | 2 (8) | 2 (6) |
| Unclear | 5 (19) | 9 (26) |
| **MSC Dose** |  |  |
| ≤ 500,000 cells | 6 (23) | 10 (29) |
| 500,000 to 1x10^6^ cells | 11 (42) | 14 (41) |
| > 1x10^6^ cells | 9 (35) | 10 (29) |
| **MSC Administration Route** |  |  |
| Intravenous | 22 (85) | 29 (85) |
| Intraperitoneal | 4 (15) | 5 (15) |

**Legend**: ^a^ For the sepsis model and resuscitation characteristics, one or more studies used multiple types of sepsis model/resuscitation method for their experiments. CLP= cecal ligation and puncture; LPS = lipopolysaccharide; D-gal = D-galactosamine; MSC = mesenchymal stromal cells.

**Supplemental Table 2. Reason for “unclear” bias rating in risk of bias assessment using the SYRCLE tool.**

| Author (year) | Sequence Generation | | Allocation Concealment | | Random Housing | | Blinding of Participants and Personnel | | Random Outcome Assessment | | Blinding of Outcome Assessors | | | | | Incomplete Outcome Data | | | |
| --- | --- | --- | --- | --- | --- | --- | --- | --- | --- | --- | --- | --- | --- | --- | --- | --- | --- | --- | --- |
|  |  |  |  |  |  |  |  |  |  |  | Mortality | | | Subjective Secondary Outcomes | | Mortality | | Secondary Outcomes | |
|  | Reported randomization but did not specify the method | Did not report randomization at all | Reported concealment but did not describe the method | Did not report a method of concealment at all | Reported randomization of housing but did not specify the method | Housing placement unreported | Insufficient detail about blinding method to permit judgement | The study did not address blinding | Reported but did not specify method of randomization for choosing animals for outcome assessment | Did not report a method of randomization for choosing animals for outcome assessment at all | Insufficient information to determine if animals were allowed to die | Mortality was assessed through surrogate endpoint, but did not report blinding at all | Mortality was assessed through surrogate endpoint, but did not report blinding in enough detail to permit judgment | Insufficient detail about blinding method to permit judgement | The study did not address blinding | N number only in methods | N number only in results | N number only in methods | N number only in results |
| Capcha et al., 2019 | Y | N | N | Y | N | Y | N | Y | N/A | N/A | N/A | N/A | N/A | N/A | N/A | N/A | N/A | N/A | N/A |
| Chao et al., 2014 | N | N | N | Y | N | Y | N | Y | N | Y | Y | N | N | N/A | N/A | N | Y | N | Y |
| Chen et al., 2019 | Y | N | N | Y | N | Y | N | Y | N | Y | N/A | N/A | N/A | N/A | N/A | N/A | N/A | Y | N |
| Chen J et al., 2021 | Y | N | N | Y | N | Y | N | Y | N | Y | N/A | N/A | N/A | N | Y | N/A | N/A | N/A | N/A |
| Chen R et al., 2021 | Y | N | N | Y | N | Y | N | Y | N | Y | Y | N | N | N/A | N/A | N/A | N/A | N/A | N/A |
| Condor et al., 2016 | Y | N | N | Y | N | Y | N | Y | N | Y | Y | N | N | N/A | N/A | N/A | N/A | Y | N |
| Huang et al., 2017 | Y | N | N | Y | N | Y | N | Y | N | Y | Y | N | N | N | Y | N/A | N/A | Y | N |
| Jerkic et al., 2020 | Y | N | N | Y | N | Y | N | Y | N/A | N/A | Y | N | N | N | Y | N | Y | N | Y |
| Laroye et al., 2018 | Y | N | Y | N | N | Y | N/A | N/A | N/A | N/A | N/A | N/A | N/A | N/A | N/A | N/A | N/A | N/A | N/A |
| Lee et al., 2017 | Y | N | N | Y | N | Y | N | Y | N | Y | N/A | N/A | N/A | N/A | N/A | N/A | N/A | N/A | N/A |
| Li et al., 2016 | Y | N | N | Y | N | Y | N | Y | N | Y | N/A | N/A | N/A | N | Y | N/A | N/A | Y | N |
| Li et al., 2012 | Y | N | N | Y | N | Y | N | Y | N | Y | Y | N | N | N/A | N/A | Y | N | Y | N |
| Li et al., 2020 | N | Y | N | Y | N | Y | Y | N | N/A | N/A | Y | N | N | N | Y | N | Y | Y | N |
| Liang et al., 2019 | Y | N | N | Y | N | Y | N | Y | N | Y | Y | N | N | N/A | N/A | N | Y | N | Y |
| Liu et al., 2021 | Y | N | N | Y | N | Y | N | Y | N | Y | N/A | N/A | N/A | N/A | N/A | N/A | N/A | N | Y |
| Long et al., 2020 | Y | N | N | Y | N | Y | N | Y | N | Y | Y | N | N | N/A | N/A | N/A | N/A | N/A | N/A |
| Song et al., 2017 | Y | N | N | Y | N | Y | N | Y | N | Y | Y | N | N | N | Y | N | Y | N | Y |
| Varkouhi et al., 2021 | Y | N | N | Y | N | Y | N | Y | N | Y | Y | Y | N | N | Y | N | Y | N | Y |
| Wang et al., 2019 | Y | N | N | Y | N | Y | N | Y | N | Y | Y | N | N | N/A | N/A | N | N | N | Y |
| Wang et al., 2022 | Y | N | N | Y | N | Y | N | Y | N | Y | Y | N | N | N/A | N/A | N | Y | N | Y |
| Wu et al., 2016 | N | Y | N | Y | N | Y | N | Y | N | Y | Y | N | N | N/A | N/A | N | Y | N | Y |
| Xu et al., 2021 | Y | N | N | Y | N | Y | N | Y | N | Y | Y | N | N | N/A | N/A | N | Y | N | N |
| Yang et al., 2015 | Y | N | N | Y | N | Y | N | Y | N | Y | N/A | N/A | N/A | N/A | N/A | N/A | N/A | N/A | N/A |
| Zeng et al., 2015 | Y | N | N | Y | N | Y | N | Y | N | Y | N | N | N | N/A | N/A | N | N | N/A | N/A |
| Zhao et al., 2014 | Y | N | N | Y | N | Y | N | Y | N | Y | Y | N | N | N | Y | N | Y | N | Y |
| Zhou et al., 2014 | Y | N | N | Y | N | Y | N | Y | N | Y | Y | N | N | N/A | N/A | N/A | N/A | N/A | N/A |

**Legend:** Y = yes; N = no; N/A = not applicable

**Supplemental Figure 1. Mortality estimates of UC-MSC treatment over time.**

**
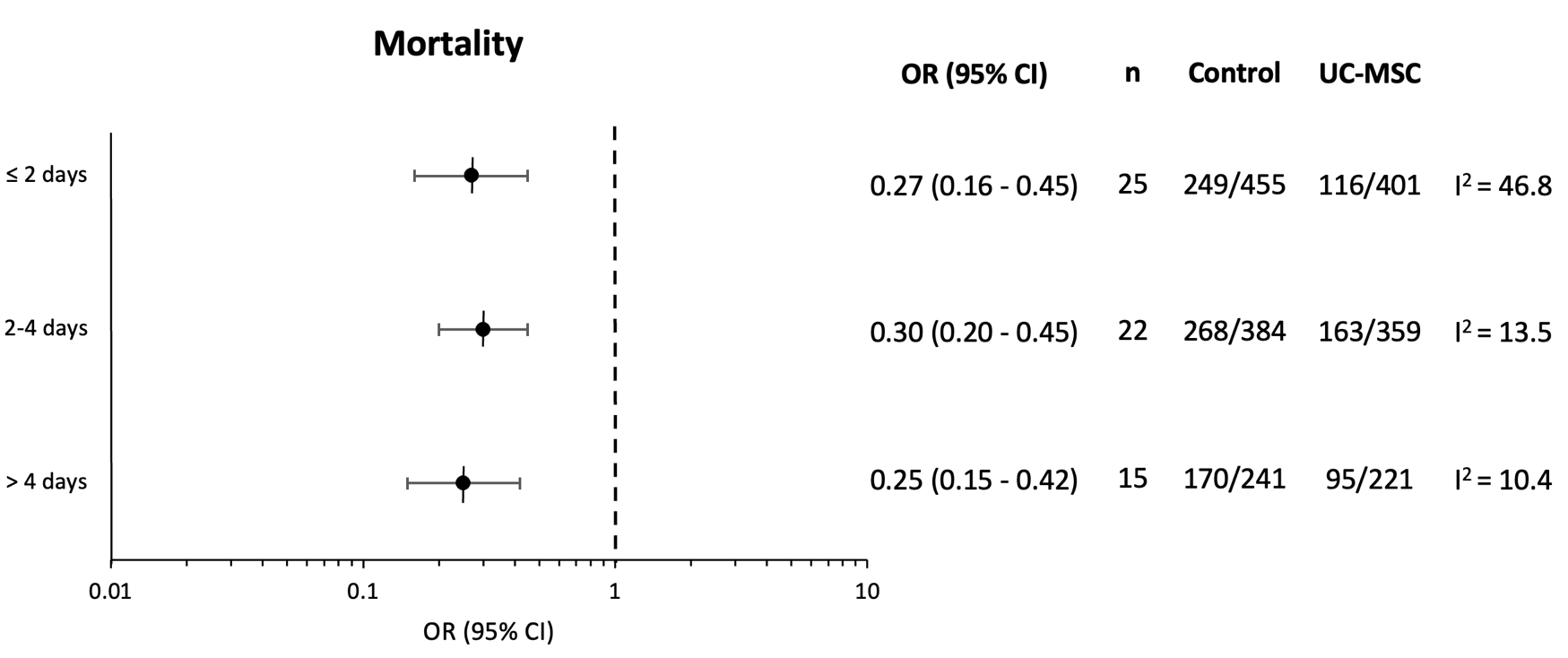
**

**Supplemental Figure 2. Overall mortality estimates of UC-MSC treatment according to pre-specified subgroups.**


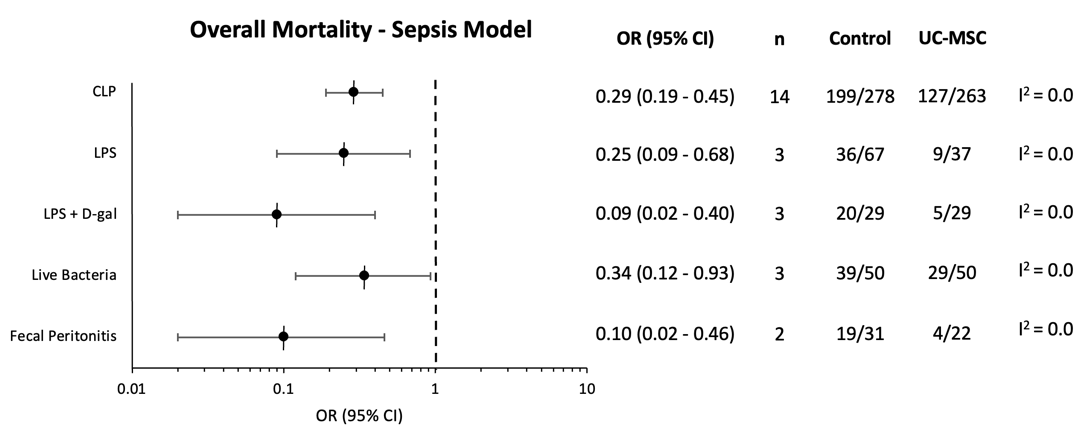

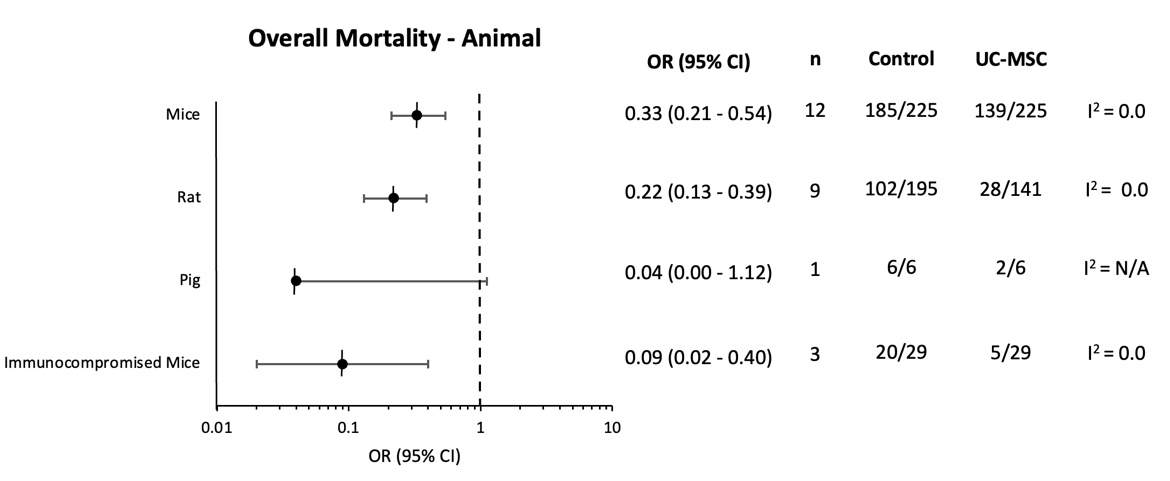


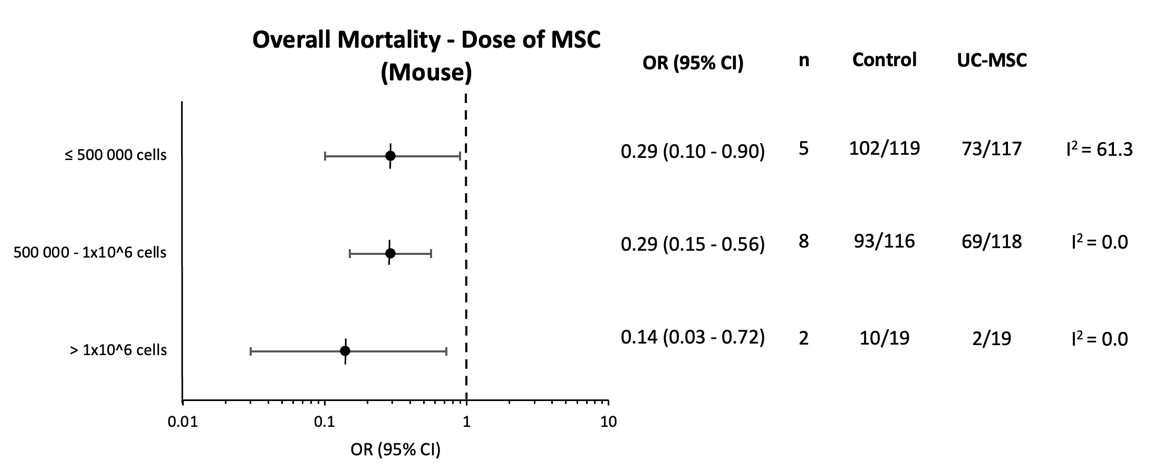

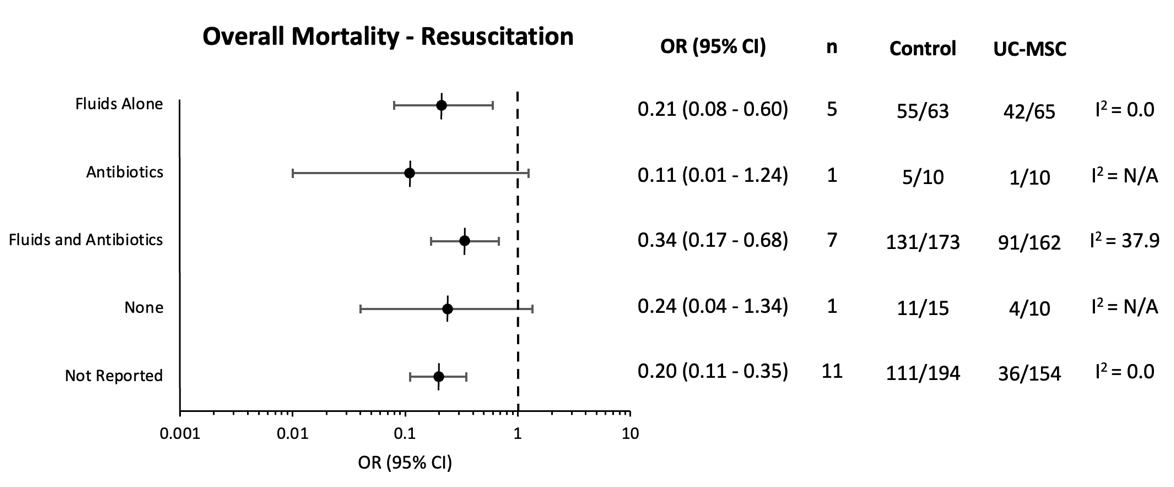


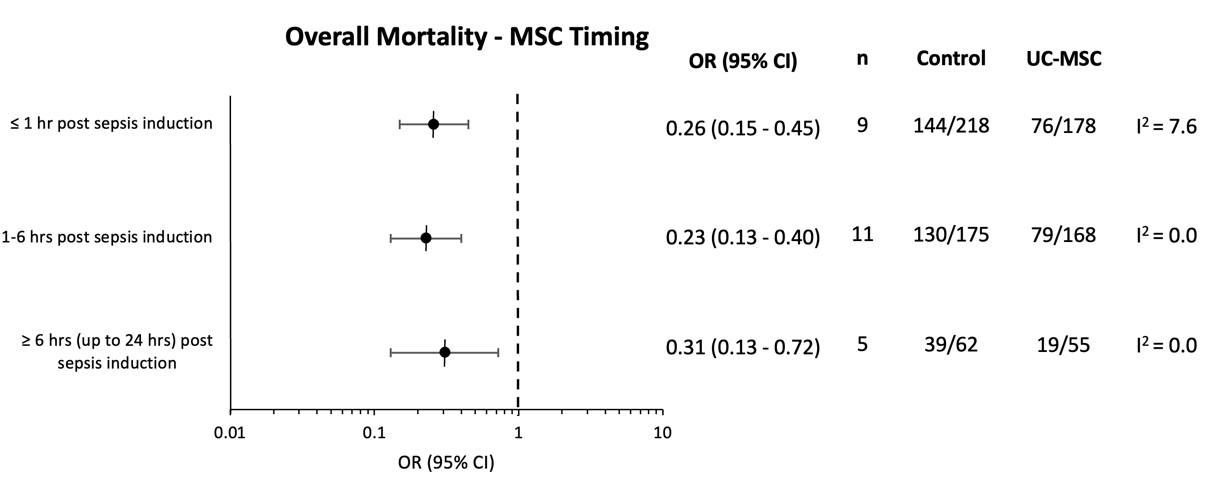

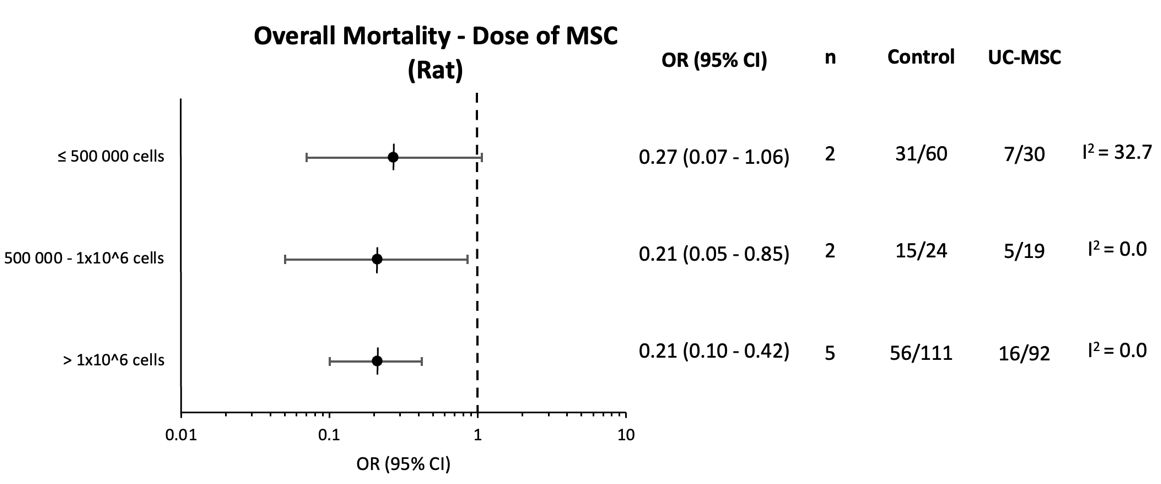


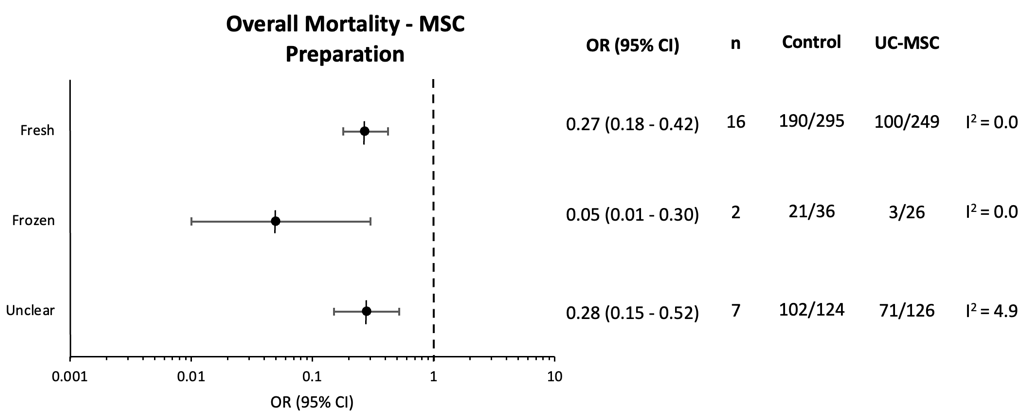


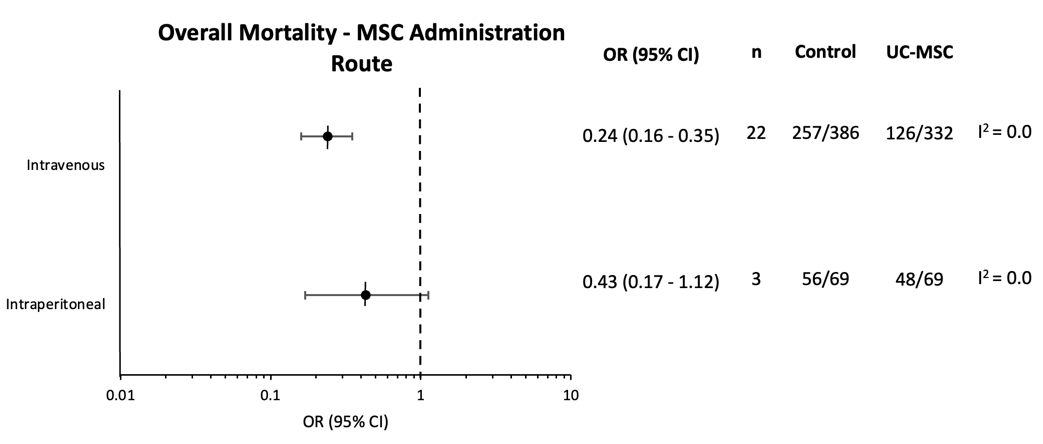


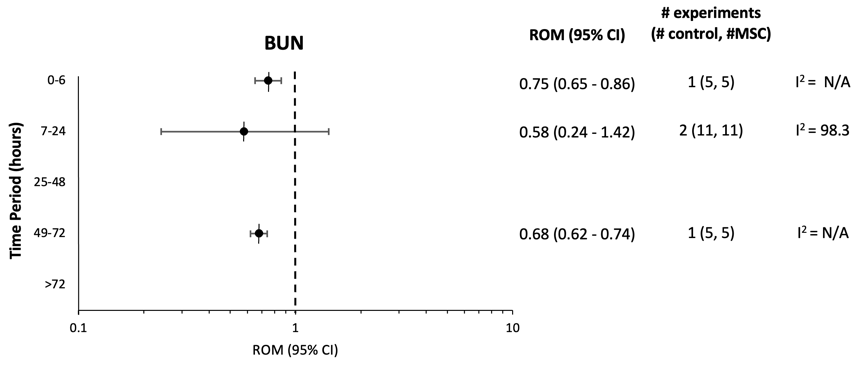

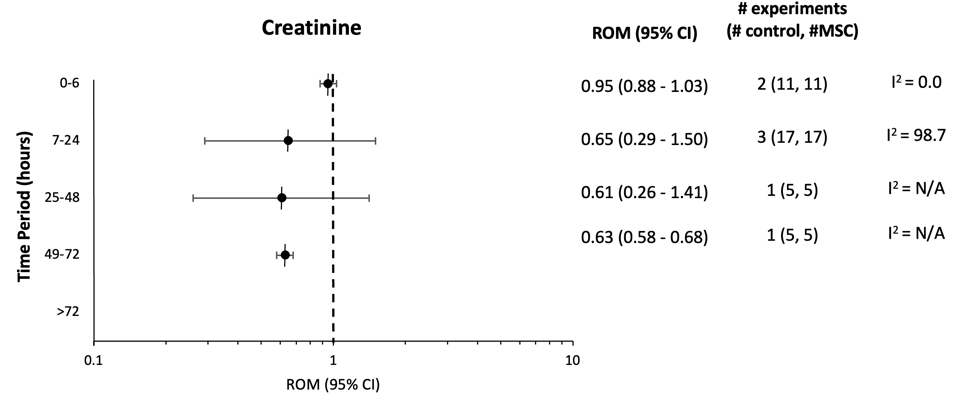
**Supplemental Figure 3. Effect of UC-MSC treatment on surrogate measures of renal dysfunction in pre-clinical models of sepsis.** Point estimates represent the ratio of means (ROM) for all data at each pre-specified time interval. The corresponding horizontal line represents the 95% confidence interval (CI).

**Supplemental Figure 4. Effect of UC-MSC treatment on surrogate measures of liver dysfunction in pre-clinical models of sepsis.** Point estimates represent the ratio of means (ROM) for all data at each pre-specified time interval. The corresponding horizontal line represents the 95% confidence interval (CI).

**
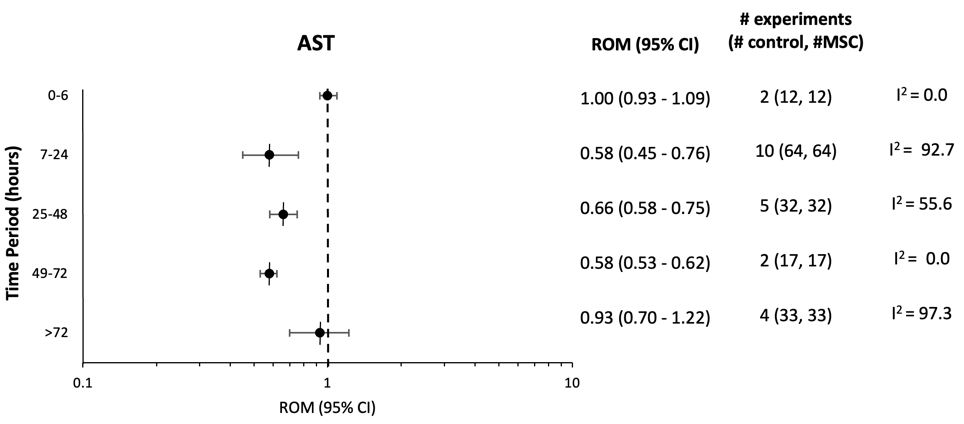

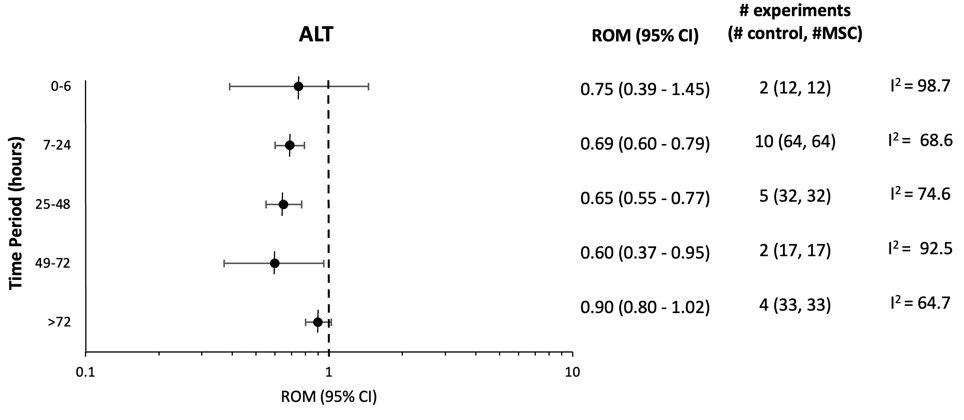
**

**Supplemental Figure 5. Effect of UC-MSC treatment on surrogate measures of pulmonary dysfunction in pre-clinical models of sepsis.** Point estimates represent the ratio of means (ROM) for all data at each pre-specified time interval. The corresponding horizontal line represents the 95% confidence interval (CI).

**Supplemental Figure 6. Effect of UC-MSC treatment on surrogate measures of cardiac dysfunction in pre-clinical models of sepsis.** Point estimates represent the ratio of means (ROM) for all data at each pre-specified time interval. The corresponding horizontal line represents the 95% confidence interval (CI).

**Supplemental Figure 7. Effect of UC-MSC treatment on coagulopathy in pre-clinical models of sepsis.** Point estimates represent the ratio of means (ROM) for all data at each pre-specified time interval. The corresponding horizontal line represents the 95% confidence interval (CI).

**Supplemental Figure 8. Effect of UC-MSC treatment on endothelial permeability in pre-clinical models of sepsis.** Point estimates represent the ratio of means (ROM) for all data at each pre-specified time interval. The corresponding horizontal line represents the 95% confidence interval (CI).

**Supplemental Figure 9. Effect of UC-MSC treatment on pathogen clearance in pre-clinical models of sepsis.** Point estimates represent the ratio of means (ROM) for all data at each pre-specified time interval. The corresponding horizontal line represents the 95% confidence interval (CI).

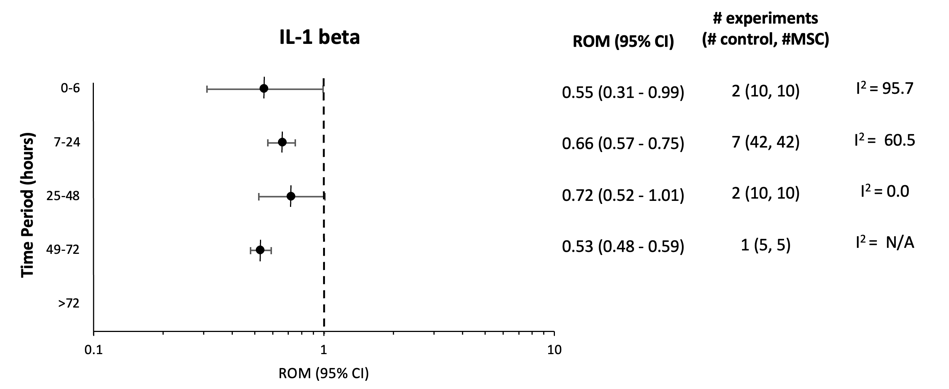

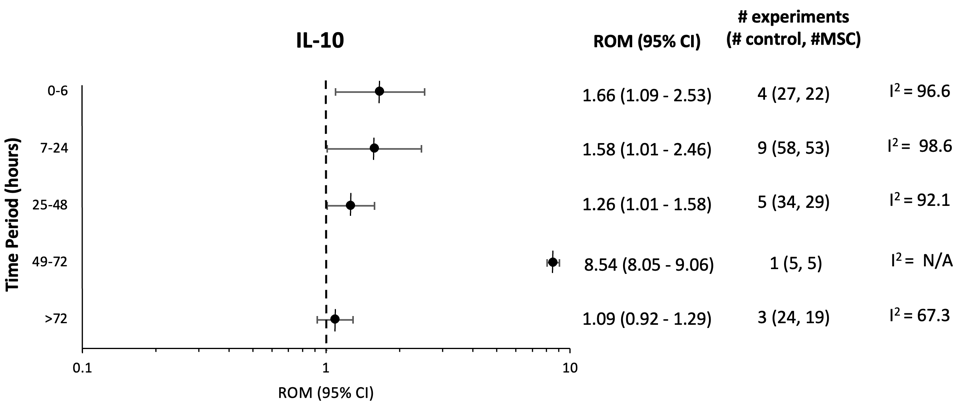

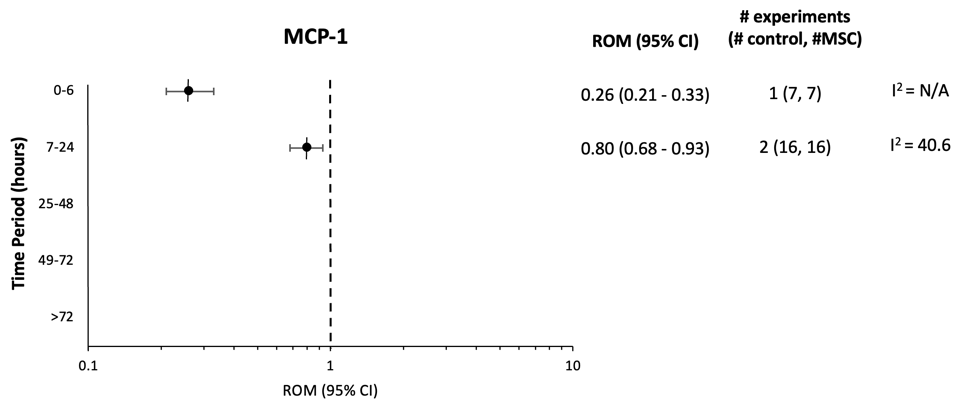

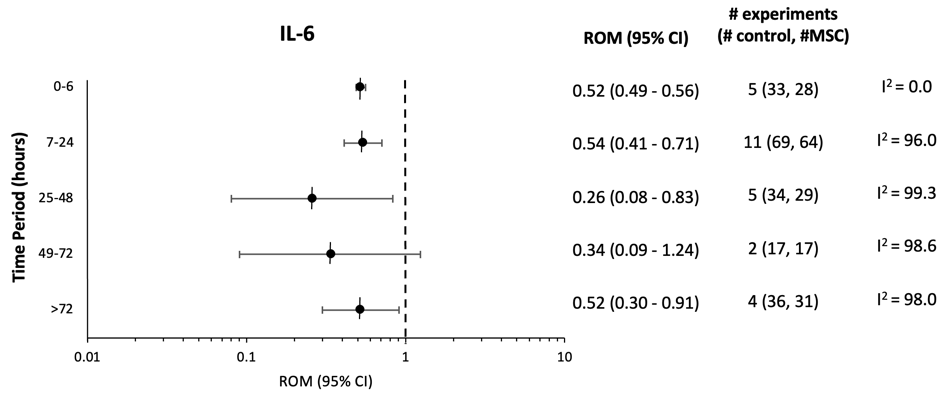

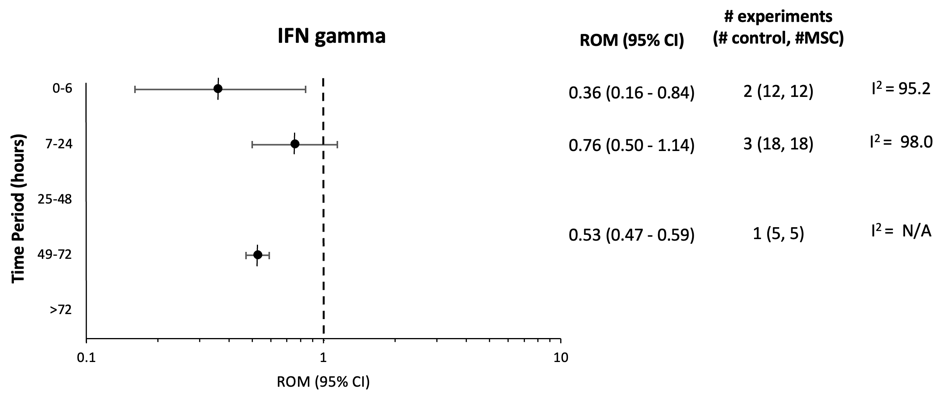

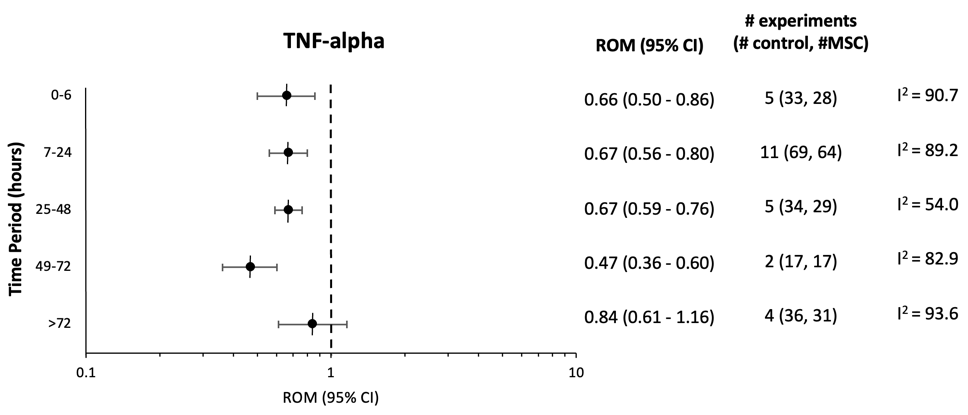
**Supplemental Figure 10. Effect of UC-MSC treatment on systemic inflammation in pre-clinical models of sepsis.** Point estimates represent the ratio of means (ROM) for all data at each pre-specified time interval. The corresponding horizontal line represents the 95% confidence interval (CI)

**Appendix: Search Strategy**

**Ovid MEDLINE(R) ALL <1946 to June 19, 2023>**

1 exp Mesenchymal Stem Cells/ 50358

2 exp Mesenchymal Stem Cell Transplantation/ 14801

3 exp Multipotent Stem Cells/ 52977

4 exp Mesenchymal Stromal Cells/ 50358

5 ((mesenchymal adj3 (stem or stroma$1 or progenitor*)) and cell$1).tw. 70572

6 (MSC or MSCs or ADMSC or ADMSCs or BM-MSC or BM-MSCs or BMD-MSC or BMD-MSCs or BMDMSC or BMDMSCs).tw. 43334

7 ((multipotent or multi-potent) adj3 (stroma$1 cell$1 or stem cell$1)).tw. 5512

8 marrow stroma$1 cell$1.tw. 7821

9 (colony-forming unit fibroblast* or CFU-F$1).tw. 944

10 exp Mesoderm/cy 7192

11 or/1-10 100563

12 Stem Cell Transplantation/ 26143

13 exp Gene Therapy/ 53723

14 Mesenchymal.tw. 150674

15 (12 or 13) and 14 2695

16 11 or 15 100843

17 exp Sepsis/ 141157

18 exp Bacteremia/ 32809

19 (sepsis* or septic* or pyaemi* or pyemi* or pyohemi*).tw. 177253

20 shock.tw. 202866

21 (fungemi* or fungaemi*or bacteremi* or bacteraemi* or endotoxemi* or endo-toxemi* or endotoxaemi* or endo-toxaemi*).tw. 19512

22 (blood adj1 poison*).tw. 120

23 ((live or viable or blood or bloodstream* or clot or clots) adj3 bacter*).tw. 14912

24 (Cecum/ or Colon,Ascending/) and ((in or su).fs. or Punctures/ or Ligation/) 3876

25 ((Cecum or coecum or caecum or cecal or coecal or caecal) adj3 (perforat* or ligat* or punctur* or injur*)).tw. 6558

26 (Colon adj1 ascend* adj3 (perforat* or ligat* or punctur* or injur*)).tw. 91

27 ((hepatic flexure or hepatic flexture) adj3 (perforat* or ligat* or punctur* or injur*)).tw. 8

28 ((right colic flexure or right colic flexture) adj3 (perforat* or ligat* or punctur* or injur*)).tw. 1

29 (proximal colon adj3 (perforat* or ligat* or punctur* or injur*)).tw. 9

30 colon ascendens stent peritonitis.tw. 83

31 (CLP or SL-CLP or CASP).tw. 10553

32 exp systemic inflammatory response syndrome/ 148946

33 ("systemic inflammatory response" or "inflammatory response syndrome" or SIRS).tw. 15139

34 exp lipopolysaccharides/ 95496

35 (lipopolysaccharide* or lipo-polysaccharide* or LPS or lipoglycan*).tw. 139481

36 exp Peritonitis/ 29038

37 peritonitis.tw. 34047

38 (exp Infection/ or exp Bacterial Infections/ or exp Inflammation/) and pp.fs. 142723

39 exp Endotoxins/ 117886

40 (endotoxin* or ETX).tw. 40177

41 or/17-40 771405

42 exp Drug Evaluation, Preclinical/ 289924

43 (preclinic* or pre-clinic*).tw. 151205

44 exp "animal experimentation"/ or exp "models, animal"/ or exp "invertebrates"/ or "Animals"/ or exp "animal population groups"/ or "chordata"/ or exp "chordata, nonvertebrate"/ or "vertebrates"/ or exp "amphibians"/ or exp "birds"/ or exp "fishes"/ or exp "reptiles"/ or "mammals"/ or "primates"/ or exp "artiodactyla"/ or exp "carnivora"/ or exp "cetacea"/ or exp "chiroptera"/ or exp "elephants"/ or exp "hyraxes"/ or exp "insectivora"/ or exp "lagomorpha"/ or exp "marsupialia"/ or exp "monotremata"/ or exp "perissodactyla"/ or exp "rodentia"/ or exp "scandentia"/ or exp "sirenia"/ or exp "xenarthra"/ or "haplorhini"/ or exp "strepsirhini"/ or exp "platyrrhini"/ or exp "tarsii"/ or "catarrhini"/ or exp "cercopithecidae"/ or exp "hylobatidae"/ or "hominidae"/ or exp "gorilla gorilla"/ or exp "pan paniscus"/ or exp "pan troglodytes"/ or exp "pongo pygmaeus"/ 7344239

45 (animals or animal or mice or mus or mouse or murine or woodmouse or rats or rat or murinae or muridae or cottonrat or cottonrats or hamster or hamsters or cricetinae or rodentia or rodent or rodents or pigs or pig or swine or swines or piglets or piglet or boar or boars or "sus scrofa" or ferrets or ferret or polecat or polecats or "mustela putorius" or "guinea pigs" or "guinea pig" or cavia or callithrix or marmoset or marmosets or cebuella or hapale or octodon or chinchilla or chinchillas or gerbillinae or gerbil or gerbils or jird or jirds or merione or meriones or rabbits or rabbit or hares or hare or diptera or flies or fly or dipteral or drosphila or drosophilidae or cats or cat or carus or felis or nematoda or nematode or nematoda or nematode or nematodes or sipunculida or dogs or dog or canine or canines or canis or sheep or sheeps or mouflon or mouflons or ovis or goats or goat or capra or capras or rupicapra or chamois or haplorhini or monkey or monkeys or anthropoidea or anthropoids or saguinus or tamarin or tamarins or leontopithecus or hominidae or ape or apes or pan or paniscus or "pan paniscus" or bonobo or bonobos or troglodytes or "pan troglodytes" or gibbon or gibbons or siamang or siamangs or nomascus or symphalangus or chimpanzee or chimpanzees or prosimians or "bush baby" or prosimian or bush babies or galagos or galago or pongidae or gorilla or gorillas or pongo or pygmaeus or "pongo pygmaeus" or orangutans or pygmaeus or lemur or lemurs or lemuridae or horse or horses or pongo or equus or cow or calf or bull or chicken or chickens or gallus or quail or bird or birds or quails or poultry or poultries or fowl or fowls or reptile or reptilia or reptiles or snakes or snake or lizard or lizards or alligator or alligators or crocodile or crocodiles or turtle or turtles or amphibian or amphibians or amphibia or frog or frogs or bombina or salientia or toad or toads or "epidalea calamita" or salamander or salamanders or eel or eels or fish or fishes or pisces or catfish or catfishes or siluriformes or arius or heteropneustes or sheatfish or perch or perches or percidae or perca or trout or trouts or char or chars or salvelinus or "fathead minnow" or minnow or cyprinidae or carps or carp or zebrafish or zebrafishes or goldfish or goldfishes or guppy or guppies or chub or chubs or tinca or barbels or barbus or pimephales or promelas or "poecilia reticulata" or mullet or mullets or seahorse or seahorses or mugil curema or atlantic cod or shark or sharks or catshark or anguilla or salmonid or salmonids or whitefish or whitefishes or salmon or salmons or sole or solea or "sea lamprey" or lamprey or lampreys or pumpkinseed or sunfish or sunfishes or tilapia or tilapias or turbot or turbots or flatfish or flatfishes or sciuridae or squirrel or squirrels or chipmunk or chipmunks or suslik or susliks or vole or voles or lemming or lemmings or muskrat or muskrats or lemmus or otter or otters or marten or martens or martes or weasel or badger or badgers or ermine or mink or minks or sable or sables or gulo or gulos or wolverine or wolverines or minks or mustela or llama or llamas or alpaca or alpacas or camelid or camelids or guanaco or guanacos or chiroptera or chiropteras or bat or bats or fox or foxes or iguana or iguanas or xenopus laevis or parakeet or parakeets or parrot or parrots or donkey or donkeys or mule or mules or zebra or zebras or shrew or shrews or bison or bisons or buffalo or buffaloes or deer or deers or bear or bears or panda or pandas or "wild hog" or "wild boar" or fitchew or fitch or beaver or beavers or jerboa or jerboas or capybara or capybaras).tw. 5415852

46 42 or 43 or 44 or 45 8483367

47 16 and 41 and 46 1789

48 limit 47 to dt=20211201-20230620 270

**Embase Classic+Embase <1947 to 2023 June 19>**

1 exp mesenchymal stem cell/ 73143

2 exp mesenchymal stem cell transplantation/ 15218

3 exp multipotent stem cell/ 6679

4 exp mesenchymal stroma cell/ 17983

5 ((mesenchymal adj3 (stem or stroma$1 or progenitor*)) and cell$1).tw. 101259

6 (MSC or MSCs or ADMSC or ADMSCs or BM-MSC or BM-MSCs or BMD-MSC or BMD-MSCs or BMDMSC or BMDMSCs).tw. 65097

7 ((multipotent or multi-potent) adj3 (stroma$1 cell$1 or stem cell$1)).tw. 7547

8 marrow stroma$1 cell$1.tw. 10807

9 (colony-forming unit fibroblast* or CFU-F$1).tw. 1562

10 exp mesoderm/ 16948

11 (cell or cytolog*).tw. 5345272

12 10 and 11 8485

13 or/1-9,12 150998

14 exp stem cell transplantation/ 191402

15 exp gene therapy/ 99145

16 Mesenchymal.tw. 213224

17 (14 or 15) and 16 20251

18 13 or 17 151629

19 exp sepsis/ 348937

20 (sepsis* or septic* or pyaemi* or pyemi* or pyohemi*).tw. 276922

21 shock.tw. 292564

22 (fungemi* or fungaemi*or bacteremi* or bacteraemi* or endotoxemi* or endo-toxemi* or endotoxaemi* or endo-toxaemi*).tw. 26470

23 (blood adj1 poison*).tw. 161

24 ((live or viable or blood or bloodstream* or clot or clots) adj3 bacter*).tw. 19419

25 (exp cecum/ or exp ascending colon/) and (exp ligation/ or exp puncture/) 1266

26 ((Cecum or coecum or caecum or cecal or coecal or caecal) adj3 (perforat* or ligat* or punctur* or injur*)).tw. 9223

27 (colon adj1 ascend* adj3 (perforat* or ligat* or punctur* or injur*)).tw. 135

28 ((hepatic flexure or hepatic flexture) adj3 (perforat* or ligat* or punctur* or injur*)).tw. 24

29 ((right colic flexure or right colic flexture) adj3 (perforat* or ligat* or punctur* or injur*)).tw. 1

30 (proximal colon adj3 (perforat* or ligat* or punctur* or injur*)).tw. 13

31 colon ascendens stent peritonitis.tw. 121

32 (CLP or SL-CLP or CASP).tw. 14799

33 exp systemic inflammatory response syndrome/ 361840

34 ("systemic inflammatory response" or "inflammatory response syndrome" or SIRS).tw. 24361

35 exp lipopolysaccharide/ 146186

36 (lipopolysaccharide* or lipo-polysaccharide* or LPS or lipoglycan*).tw. 180871

37 exp peritonitis/ 70935

38 peritonitis.tw. 50227

39 (exp Infection/ or exp bacterial infection/ or exp inflammation/) and exp pathophysiology/ 134849

40 exp endotoxin/ 46738

41 (endotoxin* or ETX).tw. 52035

42 or/19-41 1106087

43 (preclinical* or pre-clinical*).tw. 238543

44 exp animal experiment/ or exp animal model/ or exp animal/ or exp chordata/ or exp experimental animal/ or exp transgenic animal/ or exp male animal/ or exp female animal/ or exp juvenile animal/ or vertebrate/ or exp fish/ or exp amphibia/ or exp reptile/ or exp bird/ or mammal/ or exp hyrax/ or exp marsupial/ or exp monotremate/ or exp scandentia/ or placental mammals/ 33280755

45 exp bat/ or exp carnivora/ or exp cetacea/ or exp edentata/ or exp elephant/ or exp insectivora/ or exp lagomorph/ or exp rodent/ or exp sirenia/ or exp ungulate/ or primate/ or exp prosimian/ or haplorhini/ or exp tarsiiform/ or simian/ or exp platyrrhini/ or catarrhini/ 5127996

46 exp prosimian/ or haplorhini/ or exp tarsiiform/ or simian/ or exp platyrrhini/ or catarrhini/ or exp cercopithecidae/ or ape/ or exp hylobatidae/ or hominid/ or exp chimpanzee/ or exp gorilla/ or exp orang utan/ 146457

47 (animal or animals or pisces or fish or fishes or catfish or catfishes or sheatfish or silurus or arius or heteropneustes or clarias or gariepinus or fathead minnow or fathead minnows or pimephales or promelas or cichlidae or trout or trouts or char or chars or salvelinus or salmo or oncorhynchus or guppy or guppies or millionfish or poecilia or goldfish or goldfishes or carassius or auratus).tw. 1920938

48 (mullet or mullets or mugil or curema or shark or sharks or cod or cods or gadus or morhua or carp or carps or cyprinus or carpio or killifish or eel or eels or anguilla or zander or sander or lucioperca or stizostedion or turbot or turbots or psetta or flatfish or flatfishes or plaice or pleuronectes or platessa or tilapia or tilapias or oreochromis or sarotherodon or common sole or dover sole or solea or zebrafish or zebrafishes or danio or rerio or seabass or dicentrarchus).tw. 136109

49 (labrax or morone or lamprey or lampreys or petromyzon or pumpkinseed or pumpkinseeds or lepomis or gibbosus or herring or clupea or harengus or amphibia or amphibian or amphibians or anura or salientia or frog or frogs or rana or toad or toads or bufo or xenopus or laevis or bombina or epidalea or calamita or salamander or salamanders or newt or newts or triturus or reptilia or reptile or reptiles or bearded dragon or pogona or vitticeps or iguana or iguanas or lizard).tw. 152339

50 (lizards or anguis fragilis or turtle or turtles or snakes or snake or aves or bird or birds or quail or quails or coturnix or bobwhite or colinus or virginianus or poultry or poultries or fowl or fowls or chicken or chickens or gallus or zebra finch or taeniopygia or guttata or canary or canaries or serinus or canaria or parakeet or parakeets or grasskeet or parrot or parrots or psittacine or psittacines or shelduck or tadorna or goose or geese or branta or leucopsis or woodlark or lullula or flycatcher or ficedula or hypoleuca or dove or doves or geopelia or cuneata or duck or ducks).tw. 310282

51 (greylag or graylag or anser or harrier or circus pygargus or red knot or great knot or calidris or canutus or godwit or limosa or lapponica or meleagris or gallopavo or jackdaw or corvus or monedula or ruff or philomachus or pugnax or lapwing or peewit or plover or vanellus or swan or cygnus or columbianus or bewickii or gull or chroicocephalus or ridibundus or albifrons or great tit or parus or aythya or fuligula or streptopelia or risoria or spoonbill or platalea or leucorodia or blackbird or turdus or merula or blue tit or cyanistes or pigeon or pigeons or columba or pintail or anas or starling or sturnus or owl or athene noctua or pochard or ferina or cockatiel or nymphicus or hollandicus or skylark or alauda or tern or sterna or teal or crecca or oystercatcher or haematopus or ostralegus or shrew or shrews or sorex or araneus or crocidura or russula or european mole or talpa or chiroptera or bat or bats or eptesicus or serotinus or myotis).tw. 80071

52 (dasycneme or daubentonii or pipistrelle or pipistrellus or cat or cats or felis or catus or feline or dog or dogs or canis or canine or canines or otter or otters or lutra or badger or badgers or meles or fitchew or fitch or foumart or foulmart or ferrets or ferret or polecat or polecats or mustela or putorius or weasel or weasels or fox or foxes or vulpes or common seal or phoca or vitulina or grey seal or halichoerus or horse or horses or equus or equine or equidae or donkey or donkeys or mule or mules or pig or pigs or swine or swines or hog or hogs or boar or boars or porcine or piglet or piglets or sus or scrofa or llama or llamas or lama or glama or deer or deers or cervus or elaphus or cow or cows or bos taurus or bos indicus or bovine or bull or bulls or cattle or bison or bisons or sheep or sheeps or ovis aries or ovine or lamb or lambs or mouflon or mouflons or goat or goats).tw. 1610454

53 (capra or caprine or chamois or rupicapra or leporidae or lagomorpha or lagomorph or rabbit or rabbits or oryctolagus or cuniculus or laprine or hares or lepus or rodentia or rodent or rodents or murinae or mouse or mice or mus or musculus or murine or woodmouse or apodemus or rat or rats or rattus or norvegicus or guinea pig or guinea pigs or cavia or porcellus or hamster or hamsters or mesocricetus or cricetulus or cricetus or gerbil or gerbils or jird or jirds or meriones or unguiculatus or jerboa or jerboas or jaculus or chinchilla or chinchillas or beaver or beavers or castor fiber or castor canadensis or sciuridae or squirrel or squirrels or sciurus or chipmunk or chipmunks or marmot or marmots or marmota or suslik or susliks or spermophilus or cynomys or cottonrat or cottonrats or sigmodon or vole or voles or microtus or myodes or glareolus or primate or primates or prosimian or prosimians or lemur or lemurs or lemuridae or loris or bush baby or bush babies or bushbaby or bushbabies or galago or galagos or anthropoidea or anthropoids).tw. 4490656

54 (simian or simians or monkey or monkeys or marmoset or marmosets or callithrix or cebuella or tamarin or tamarins or saguinus or leontopithecus or squirrel monkey or squirrel monkeys or saimiri or night monkey or night monkeys or owl monkey or owl monkeys or douroucoulis or aotus or spider monkey or spider monkeys or ateles or baboon or baboons or papio or rhesus monkey or macaque or macaca or mulatta or cynomolgus or fascicularis or green monkey or green monkeys or chlorocebus or vervet or vervets or pygerythrus or hominoidea or ape or apes or hylobatidae or gibbon or gibbons or siamang or siamangs or nomascus or symphalangus or hominidae or orangutan or orangutans or pongo or chimpanzee or chimpanzees or pan troglodytes or bonobo or bonobos or pan paniscus or gorilla or gorillas or troglodytes).tw. 195767

55 or/43-54 33958211

56 18 and 42 and 55 4706

57 limit 56 to dc=20211201-20230620 958

**# Web of Science Search Strategy (v0.1)**

# Database: Web of Science Core Collection

# Entitlements:

- WOS.SCI: 1900 to 2023

- WOS.AHCI: 1975 to 2023

- WOS.ESCI: 2005 to 2023

- WOS.ISTP: 1990 to 2023

- WOS.SSCI: 1900 to 2023

- WOS.ISSHP: 1990 to 2023

# Searches:

1: TS= (Mesenchymal Stem Cells) OR TS= (Mesenchymal Stem Cell Transplantation) OR TS= (Multipotent Stem Cells) OR TS=(Mesenchymal Stromal Cells) Results: 138108

2: TS= ((((mesenchymal NEAR/3 (stem or stroma$ or progenitor*))))) OR TS= (((multipotent NEAR/3 (stroma$ or stem)))) OR TS= (((multi-potent NEAR/3 (stroma$ or stem)))) OR TS= (((MSC or MSCs or ADMSC or ADMSCs or BM-MSC or BM-MSCs or BMD-MSC or BMD-MSCs or BMDMSC or BMDMSCs))) OR TS= ((((marrow stroma cell or marrow stromal cell or marrow stroma cells or marrow stromal cells)))) OR TS= (((colony-forming unit fibroblast* or CFU-F or CFU-Fs))) OR TS= (((mesoderm and cytolog*))) Results: 152984

3: #2 OR #1 Results: 175094

4: TS= (((Stem Cell Transplantation OR Gene Therapy))) AND TS= (Mesenchymal) Results: 36195

5: #4 OR #3 Results: 179849

6: TS= (Sepsis) OR TS= (Bacteremia) OR TS= (((sepsis* or septic* or pyaemi* or pyemi* or pyohemi*))) Results: 245578

7: TS= (shock) OR TS= (((fungemi* or fungaemi*or bacteremi* or bacteraemi* or endotoxemi* or endo-toxemi* or endotoxaemi* or endo-toxaemi*))) OR TS= (((blood NEAR/1 poison*))) Results: 497336

8: TS= ((((live or viable or blood or bloodstream* or clot or clots) NEAR/3 bacter*))) Results: 24439

9: TS= ((((Cecum or coecum or caecum or cecal or coecal or caecal) NEAR/3 (perforat* or ligat* or punctur* or injur*)))) Results: 6696

10: TS= ((((colon NEAR/1 ascend*) NEAR/3 (perforat* or ligat* or punctur* or injur*)))) OR TS= (("hepatic flexture" NEAR/3 (perforat* or ligat* or punctur* or injur*))) OR TS= (("hepatic flexure" NEAR/3 (perforat* or ligat* or punctur* or injur*))) OR TS= (("right colic flexure" NEAR/3 (perforat* or ligat* or punctur* or injur*))) OR TS= (("right colic flexture" NEAR/3 (perforat* or ligat* or punctur* or injur*))) OR TS= (("proximal colon" NEAR/3 (perforat* or ligat* or punctur* or injur*))) OR TS= ((colon ascendens stent peritonitis)) OR TS= (((CLP or SL-CLP or CASP))) OR TS= ((systemic inflammatory response syndrome)) OR TS= ((("systemic inflammatory response" or "inflammatory response syndrome" or SIRS))) OR TS= ((lipopolysaccharide)) OR TS= (((lipopolysaccharide* or lipo-polysaccharide* or LPS or lipoglycan*))) OR TS= ((peritonitis)) OR TS= (((endotoxin* or ETX))) Results: 300396

11: TS= ((Infection* or inflammation*)) AND TS= (pathophysiology) Results: 34275

12: #11 OR #10 OR #9 OR #8 OR #7 OR #6 Results: 981913

13: #12 AND #5 Results: 4065

14: TS= ((((animal$ or chordata or vertebrate* or fish or fishes or amphibian* or amphibium* or reptile$ or bird$ or mammal* or dog or dogs or canine$ or cat or cats or hyrax* or marsupial* or monotrem* or scandentia or bat or bats or carnivor* or cetacea or edentata* or elephant* or insect or insects or insectivore or lagomorph* or rodent* or mouse or mice or murine or murinae or muridae or rat or rats or pig or pigs or piglet$ or swine or rabbit$ or sheep$ or goat$ or horse$ or equus or cow or cows or cattle or calf or calves or bovine or sirenia or ungulate$ or primate$ or prosimian* or haplorhini* or tarsiiform* or simian*or platyrrhini or catarrhini or cercopithecidae or ape or apes or hylobatidae or hominid* or chimpanzee* or gorilla* or orangutan* or monkey or monkeys or ape or apes)))) Results: 7545819

15: TS= ((((preclinic* or pre-clinic*)))) Results: 163682

16: #14 OR #15 Results: 7632880

17: #16 AND #13 Results: 2295

18: #16 AND #13 Timespan: 2021-11-01 to 2023-06-20 Results: 414
